# Antimicrobial resistance genotypes and phenotypes of *Campylobacter jejuni* isolated in Italy from humans, birds from wild and urban habitats, and poultry

**DOI:** 10.1371/journal.pone.0223804

**Published:** 2019-10-11

**Authors:** Francesca Marotta, Giuliano Garofolo, Lisa di Marcantonio, Gabriella Di Serafino, Diana Neri, Romina Romantini, Lorena Sacchini, Alessandra Alessiani, Guido Di Donato, Roberta Nuvoloni, Anna Janowicz, Elisabetta Di Giannatale

**Affiliations:** 1 National Reference Laboratory for *Campylobacter*, Istituto Zooprofilattico Sperimentale dell’Abruzzo e del Molise “G. Caporale”, Teramo, Italy; 2 Department of Veterinary Sciences, University of Pisa, Pisa, Italy; Panstwowy Instytut Weterynaryjny - Panstwowy Instytut Badawczy w Pulawach, POLAND

## Abstract

*Campylobacter jejuni*, a common foodborne zoonotic pathogen, causes gastroenteritis worldwide and is increasingly resistant to antibiotics. We aimed to investigate the antimicrobial resistance (AMR) genotypes of *C*. *jejuni* isolated from humans, poultry and birds from wild and urban Italian habitats to identify correlations between phenotypic and genotypic AMR in the isolates. Altogether, 644 *C*. *jejuni* isolates from humans (51), poultry (526) and wild- and urban-habitat birds (67) were analysed. The resistance phenotypes of the isolates were determined using the microdilution method with EUCAST breakpoints, and AMR-associated genes and single nucleotide polymorphisms were obtained from a publicly available database. Antimicrobial susceptibility testing showed that *C*. *jejuni* isolates from poultry and humans were highly resistant to ciprofloxacin (85.55% and 76.47%, respectively), nalidixic acid (75.48% and 74.51%, respectively) and tetracycline (67.87% and 49.02%, respectively). Fewer isolates from the wild- and urban-habitat birds were resistant to tetracycline (19.40%), fluoroquinolones (13.43%), and quinolone and streptomycin (10.45%). We retrieved seven AMR genes (*tet* (O), *cmeA*, *cmeB*, *cmeC*, *cmeR*, *blaOXA-61* and *blaOXA-184*) and *gyrA*-associated point mutations. Two major B-lactam genes called *blaOXA-61* and *blaOXA-184* were prevalent at 62.93% and 82.08% in the poultry and the other bird groups, respectively. Strong correlations between genotypic and phenotypic resistance were found for fluoroquinolones and tetracycline. Compared with the farmed chickens, the incidence of AMR in the C. *jejuni* isolates from the other bird groups was low, confirming that the food-production birds are much more exposed to antimicrobials. The improper and overuse of antibiotics in the human population and in animal husbandry has resulted in an increase in antibiotic-resistant infections, particularly fluoroquinolone resistant ones. Better understanding of the AMR mechanisms in *C*. *jejuni* is necessary to develop new strategies for improving AMR programs and provide the most appropriate therapies to human and veterinary populations.

## Introduction

*Campylobacter jejuni* infections are one of the most prevalent and widespread causes of bacterial diarrhoeal disease in humans. Over the last 10 years, the incidence and prevalence of campylobacteriosis has increased in both developed and developing countries, with about 500 million cases of gastroenteritis reported annually [1]. In the European Union, campylobacteriosis is considered the most frequent foodborne infection, with more than 240,000 confirmed human cases per year [2]. Most of the cases are self-limiting with symptoms such as fever, abdominal cramping and bloody diarrhoea. Rarely, the infection might lead to post-infectious neurological complications including Guillain-Barrè and Miller-Fischer syndromes. *Campylobacter* infections can also predispose people to gastrointestinal autoimmune disorders like celiac disease and inflammatory bowel disease [3]. *Campylobacter* transmission occurs mainly from exposure to farm animals with such infections, with subsequent passage through the food chain to retail food products [4, 5]. Poultry animals are considered the major infection reservoir and humans most frequently become infected by handling raw, contaminated chicken and turkey meat [4, 6]. Other food products, including beef, pork, lamb, unpasteurized milk, untreated water and seafood are also considered risk factors for campylobacteriosis [7–9]. Although *Campylobacter* enteritis is self-limiting and antibiotic treatment is usually not indicated [10], in some cases the illness can progress to bacteraemia or become an extraintestinal infection and require antimicrobial therapy, especially in immunocompromised patients [11, 12]. In such cases, the drugs of choice are macrolides and fluoroquinolones, the latter of which is the last class of antimicrobials in common use for treating all diarrheal illnesses, including traveller’s diarrhoea. However, over-use of antimicrobials in the human population and in food animals has increased the number of antibiotic-resistant infections, especially fluoroquinolone-resistant ones [13]. This is a problem because campylobacteriosis is clinically indistinguishable from the gastrointestinal infections caused by other bacterial pathogens. Consequently, the empirical use of fluoroquinolones for treating gastrointestinal infections promotes antibiotic resistance to this class of molecules.

Tetracycline and beta-lactam antimicrobials are also used to treat intestinal infections but they are not generally recommended for treating *Campylobacter* infections [14–17]. Gentamycin, however, shows potent *in vitro* activity and may be considered as an alternative treatment. *C*. *jejuni* is naturally transformable, making the acquisition of antibiotic resistant genes from other organisms likely [13]. The genetic determinants of antibiotic resistance in *C*. *jejuni*, which are chromosomally or plasmid encoded, comprise both endogenous and acquired genes [13]. In general, the different antibiotic resistance mechanisms can be summarised as follows: modification of the antimicrobial target and/or its expression (e.g., DNA gyrase mutations), inability of the antibiotic to reach its target (e.g., upregulated expression of the major outer membrane protein), antibiotic efflux (e.g., multidrug efflux pumps such as CmeABC) and modification or inactivation of the antibiotic (e.g., beta-lactam production) [13]. The different mechanisms involved in antibiotic resistance in *C*. *jejuni* are often synergic. Trends in antimicrobial resistance (AMR) have shown a clear correlation related to the use of antibiotics in the animal production industry, and antibiotic-resistant *Campylobacter* strains have been isolated from humans [17]. Some studies have supported the hypothesis that resistance patterns in poultry could be used as predictors of human resistance patterns, particularly for fluoroquinolones [18, 19]. The increment of resistant strains to commonly used antibiotics in campylobacteriosis makes it necessary the research a more reliable methods in order to investigate antimicrobial susceptibility as well as alternatives therapies [16].

These indications emphasizes the need for improved surveillance and data sharing, confirming the importance of rapid and reproducible methods predicting resistance phenotypes and defining resistance mechanism for surveillance diagnostics. The objectives of this study were to identify AMR genotypes in *C*. *jejuni* isolated from humans, poultry and birds from wild and urban environments, and to assess whether any correlations exist between phenotypic and genotypic resistance in the isolates.

## Materials and methods

### Sample selection and experimental design

Altogether, 644 *C*. *jejuni* strains from the collection at the National Reference Laboratory for *Campylobacter* (NRL, http://www.izs.it/IZS/Eccellenza/Centri_nazionali/LNR_-_Campylobacter) were selected for this study. The collection comprises 51 strains isolated from humans, 246 isolated from retail chicken meat, 280 strains isolated from broiler chickens and 67 wildlife strains isolated from birds living in wild and urban habitats. All the strains were isolated by different monitoring and surveillance plans. The human isolates were from acute campylobacteriosis cases collected in Italy during 2015–2017. The food-related isolates were obtained from a nationwide monitoring study during the one-year period from 2015–2016. The isolates from farmed animals, which came from another nationwide monitoring plan, represent 85% of the intensive broiler production facilities in Italy during the one year period from 2015–2016. The wildlife strains were isolated via passive surveillance monitoring by the Istituti Zooprofilattici Sperimenatali (IIZZSS) network during 2015–2017. The *C*. *jejuni* isolates from two greenfinches (*Chloris chloris*), one whitewagtail (*Motacilla Alba*), one owl (*Asio otus*) and two mallards (*Anas platyrhynchos*) represent birds from wild habitats. The remaining *C*. *jejuni* isolates collected from 47 pigeons (*Columba livia*), six magpies (*Pica pica*), six crows (*Corvus frugilegus*), one pheasant (*Phasianus colchicus*) and one starling (*Sturnus vulgaris*) represent birds from urban habitats.

### Microbiological analyses and antimicrobial susceptibility tests

The isolates were grown on Columbia blood agar and incubated at 42°C for 48 h in a microaerophilic atmosphere. After preliminary phenotypic characterization, the resultant colonies were confirmed to be thermotolerant *C*. *jejuni* using a multiplex PCR, as described by Wang et al. [20], and by a simplex PCR, as described by Di Giannatale et al. [21]. The primers list is shown in Table 1. DNA was extracted using the Maxwell 16 Tissue DNA Purification Kit (Promega Corp., Madison, WI) according to the manufacturer’s instructions. Antimicrobial susceptibility tests on the isolates were performed using the microdilution method to determine the minimum inhibitory concentrations (MICs) of streptomycin (S), ciprofloxacin (cip), tetracycline (Te), gentamicin (G), erythromycin (E) and nalidixic acid (NA), following the harmonised rules for the monitoring and reporting of AMR in Europe (Commission Implementing Decision 2013/652/EC). Briefly, the colonies were grown on Columbia agar for 24 h and then inoculated into Mueller Hinton Broth supplemented with blood (Oxoid, Basingstoke, UK). Then, using the Sensititre® system (Thermo Fisher Scientific, Dardilly, France) the broths were separately dispensed into Eucamp2 microtiter plates (Thermo Fisher Scientific) containing known scalar concentrations of the following antibiotics: S (0.25–16 *μ*g/mL), cip (0.12–16 *μ*g/mL), Te (0.5–64 *μ*g/mL), G (0.12–16 *μ*g/mL) E (1–128 *μ*g/mL) and NA (1–64 *μ*g/mL). After inoculation, the plates were incubated at 42°C in a microaerophilic atmosphere for 24 h and then screened. To evaluate the MICs of the isolates, Swin v3.3 Software (Thermo Fisher Scientific) was used in accordance with the epidemiological cutoff values (ECOFFs) as defined by EUCAST (European Committee on antimicrobial breakpoints) (www.eucast.org) to interpret their antimicrobial susceptibilities. We included the *C*. *jejuni* NCTC 11351 reference strain to normalise the MIC tests.

**Table 1:** List of primers used for PCR.

	Multiplex PCR primers	Sequence (5´-3´)	reference
***C*.*jejuni***	CJF (25 pm)	ACTTCTTTATTGCTTGCTGC	**[20]**
	CJR (25 pm)	GCCACAACAAGTAAAGAAGC	**[20]**
***C*.*coli***	CCF (50 pm)	GTAAAACCAAAGCTTATCGTG	**[20]**
	CCR (50 pm)	TCCAGCAATGTGTGCAATG	**[20]**
***C*.*lari***	CLF (25 pm)	TAGAGAGATAGCAAAAGAGA	**[20]**
	CLR (25 pm)	TACACATAATAATCCCACCC	**[20]**
***C*.*fetus***	CFF (50 pm)	GCAAATATAAATGTAAGCGGAGAG	**[20]**
	CFR (50 pm)	TGCAGCGGCCCCACCTAT	**[20]**
***C*.*upsaliensis***	CUF (100 pm)	AATTGAAACTCTTGCTATCC	**[20]**
	CUR (100 pm)	TCATACATTTTACCCGAGCT	**[20]**
	**Simplex PCR primers**	**Sequence (5´-3´)**	
***C*.*jejuni***	P3Fs (50 pm)	GGAAAAACAGGCGTTGTGGGGG	**[21]**
	P3Rs (50 pm)	CCGAAGAAGCCATCATCGCACC	**[21]**

### Identification of antibiotic resistance genes

*C*. *jejuni* genome assemblies were searched for the presence of genomic AMR traits. AMR genes were identified *in silico* using ABRicate v. 0.8 (https://github.com/tseemann/abricate/) and by querying the publicly available Comprehensive Antibiotic Resistance Database [22]http://cge.cbs.dtu.dk/services/ResFinder/. Assemblies were annotated using Prokka v1.13 [23] and *gyrA* sequences were extracted using the query_pan_genome function in Roary v3.12.0 [24]. *gyrA* genes were aligned using Uniprot UGENE v1.18.0 [25], from which the gene variants were identified. Only mutations in the quinolone resistance-determining region (QRDR) of *gyrA* were regarded to be the determinants of resistance, as only these loci have been linked with phenotypic resistance to quinolones. In particular, for *gyrA*, we analysed the amino acid changes at position 86.

### Correlations between phenotypic and genotypic susceptibility to antimicrobials

Correlations between the resistance phenotypes obtained from the Sensititre system and the genetic resistant determinants obtained from the genomic information available at the NRL for *Campylobacter* for the various monitoring systems were determined for the aforementioned antimicrobials. Specifically, each interpretation for a given phenotypic antibiotic result was manually compared with the presence or absence of the known corresponding resistance gene or with specific mutations, and the percentage correlation between the resistance phenotype and genotype was calculated.

## Results

### Antimicrobial resistance phenotypes

**Fig 1:**
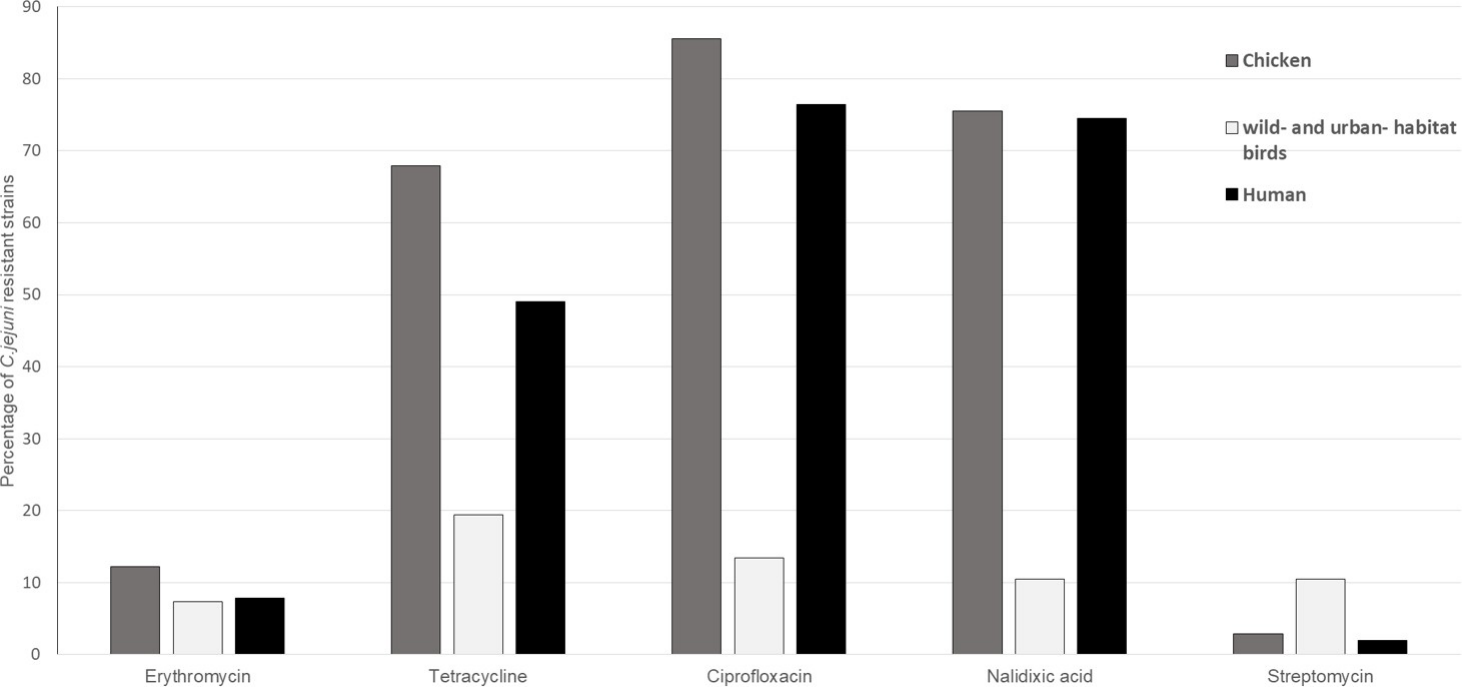
Percentage of *C*. *jejuni* strains isolated from chickens, birds from wild and urban habitats, and from humans showing resistance to antimicrobials (shown on the x-axis).

Quinolone resistance was prevalent in *C*. *jejuni* from humans, with levels of 76.47% and 74.51% for ciprofloxacin and nalidixic acid, respectively. Ciprofloxacin and nalidixic acid resistance was common in a large portion of the strains from poultry (85.55% and 75.48%, respectively). Tetracycline resistance was also evident in a large percentage of strains, with levels of 49.02% for human isolates and 67.87% for poultry. In contrast, almost all the isolates from humans and poultry were susceptible to aminoglycosides (gentamicin and streptomycin) (Table 2). The antimicrobial test results for the different groups of strains are shown in Fig 1 and Table 2.

**Table 2:** Percentage of *C*. *jejuni* isolates from humans, poultry and birds from wild and urban habitats displaying different antimicrobial susceptibility levels.

Source	Erythromycin			Gentamycin			Tetracycline			Ciprofloxacin			Nalidixic acid			Streptomycin		
	R	I	S	R	I	S	R	I	S	R	I	S	R	I	S	R	I	S
Human	7.84	29.41	62.75	1.96	0.00	98.04	49.02	0.00	50.98	76.47	0.00	23.53	74.51	0.00	25.49	1.96	5.88	92.16
Chicken—Total	12.17	61.98	25.86	1.52	0.19	98.29	67.87	2.47	29.66	85.55	0.00	14.45	75.48	0.00	24.52	2.85	15.78	81.37
Chicken—Animals	11.07	57.86	31.07	1.07	0.36	98.57	72.86	2.50	24.64	87.86	0.00	12.14	77.86	0.00	22.14	2.14	11.79	86.07
Chicken—Food	13.41	66.67	19.92	2.03	0.00	97.97	62.20	2.44	35.37	82.93	0.00	17.07	72.76	0.00	27.24	3.66	20.33	76.02
Wild and Urban Birds	7.46	82.09	10.45	2.99	0.00	97.01	19.40	1.49	79.10	13.43	0.00	86.57	10.45	0.00	89.55	10.45	7.46	82.09

R = resistant; S = sensitive; I = intermediate

Conversely, tetracycline resistance was seen more frequently in isolates from the wild and urban habitat birds (19.40%) compared with ciprofloxacin (13.43%), nalidixic acid (10.45%) and streptomycin (10.45%) (Fig 1, Table 2). In the urban habitat, nine pigeon-isolated strains showed tetracycline resistance, six showed ciprofloxacin resistance, five showed nalidixic acid resistance, five showed streptomycin resistance, three showed erythromycin resistance and two showed gentamycin resistance. While one pheasant isolate was resistant to erythromycin, tetracycline, ciprofloxacin and nalidixic acid, one isolate from the six crows was resistant to tetracycline and streptomycin and one isolate from the six magpies was resistant to nalidixic acid and streptomycin. Interestingly, with the birds from wild habitats, one strain from the whitewagtail showed resistance to erythromycin and tetracycline, while the two strains isolated from greenfinches were resistant to tetracycline and ciprofloxacin. In the latter cases, no isolates showed resistance to gentamycin, nalidixic acid and streptomycin.

It is worth noting that the strains isolated from birds inhabiting urban and wild environments, which amounted to 82.09% of the total, showed intermediate levels of susceptibility to erythromycin (Table 2).

The multidrug resistance (MDR) profiles for *C*. *jejuni* are shown in Table 3. We identified six *C*. *jejuni-*specific antimicrobial resistance profiles. At 47.90% and 17.76%, the TeCipNa MDR category was the most common for the *C*. *jejuni* strains isolated from poultry and humans, respectively. Interestingly, isolates from the birds belonging to the wild and urban environments dominated the EGTeCipNaS and ETeCipNaS MDR categories compared with the other isolate groups (poultry and human isolates) (Table 3).

**Table 3:** Percentage of antimicrobial multi-resistance patterns among *C*. *jejuni* from chickens, birds from wild and urban habitats and humans.

Antibiotic resistance pattern	Chickens–Total (%)	Birds from wild and urban habitats (%)	Humans (%)
EGTeCipNaS (n = 6)	0.38	1.49*	0.00
ETeCipNaS (n = 5)	0.90	1.49*	0.00
ETeCipNa (n = 4)	8.70**	2.98	0.00
TeCipNaS (n = 4)	1.30	1.49^¶^	1.96***
TeCipNa (n = 3)	47.90^≈^	1.49	17.76
CipNaS (n = 3)	0.00	0.00	1.96

* t-test p<0.001: birds from wild and urban habitats vs. chickens

** t-test p<0.001: chickens vs. birds from wild and urban habitats

^¶^ t-test p<0.001: birds from wild and urban habitats vs. chickens

*** t-test p<0.001: humans vs. chickens and birds from wild and urban habitats

^≈^ t-test p<0.001: humans vs. chickens and birds from wild and urban habitats

### Detection of resistance genes and mutations, and concordance between resistance phenotypes and genotypes

**Fig 2:**
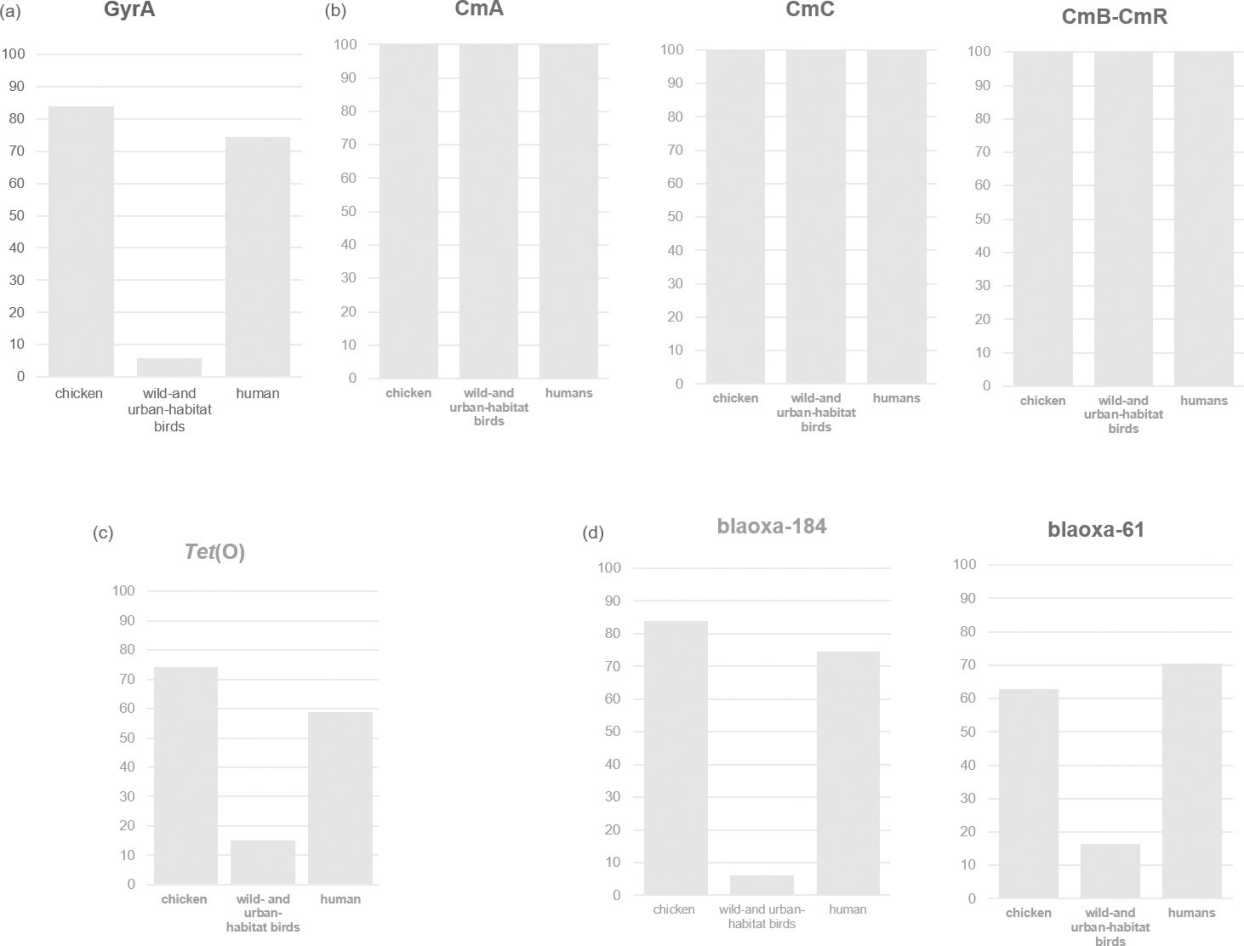
Percentage of isolates harbouring *gyr*A and *tet* (O) genes and the multi-drug resistance-related cmeABC operon, and the percentage of B-lactam genes in the resistant isolates.

All isolates showing resistance to both ciprofloxacin and nalidixic acid were screened for point mutations within *gyr*A. With the exception of eight isolates from chickens that carried the A256G point mutation, which produces a T86V substitution, the remaining isolates from chickens, humans and the birds from wild habitats possessed the C257T point mutation, resulting in a T86I substitution in GyrA, a known quinolone resistance mutation (Table 4). The *gyr*A gene was detected in 83.84% of the isolates from the chickens, in 5.97% of the isolates from birds inhabiting wild and urban areas and in 74.51% of the isolates from humans (Fig 2A). Strong correlations were found for the chicken and human isolates for phenotypic and genotypic resistance, with a high level of concordance (96.22% and 97.43%) for the two resistance rates, respectively. The CmeABC multidrug efflux pump and its CmeR regulator, which together act as a major efflux pump mechanism conferring resistance to a wide range of antimicrobials, were both identified in every strain we analysed (Fig 2B). In the tetracycline-resistant isolates from this study, the *tet* (O) gene was detected in 74.33% of the chicken isolates, 56.86% of the human isolates and 14.92% of all the isolates from birds (Fig 2C). The correlation percentages between the phenotypes (resistant and susceptible) and genotypes were 93.27% for chickens, 92.30% for birds from wild and urban habitats and 88% for humans (Table 4). Beta-lactam resistance-encoding genes (*blaOXA-61* and *blaOXA-184*) [26] were present at different levels in the analysed species. *blaOXA*-61 was detected in 70.59% of the human strains, 62.14% of the chicken strains and 17.14% of the strains from the birds from wild and urban habitats (Fig 2D). In contrast, *blaOXA-184* was detected in 11.76% of the human strains, 31.56% of the chicken strains and 82.08% of the strains from the birds from wild and urban habitats (Fig 2D). Strong correlations between phenotypic and genotypic resistance were found for fluoroquinolones and tetracycline.

**Table 4:** Correlations between resistance phenotypes and genotypes among *C*. *jejuni* isolates.

Drug class	drug (s) tested	species	no. of isolates with R phenotype	Presence of resistance genes or mutations corresponding to resistance phenotype (no. of isolates)	Correlation between genotypes and phenotype (%)
Tetracycline	Te	Chickens	n = 357	*tet* (O) (n = 333)	93.27
		Wild birds	n = 13	*tet* (O) (n = 12)	92.30
		Humans	n = 25	*tet* (O) (n = 22)	88
Quinolones, fluoroquinolones	Cip, NA	Chicken	n = 450	GyrA T86I (n = 433)—GyrA T86V (n = 8)	96.22
		Wild birds	n = 9	GyrA T86I (n = 4)	44.44
		Humans	n = 39	GyrA T86I (n = 38)	97.43

## Discussion

The increasing trend of drug resistance, particularly MDR, to the major antibiotics currently in use among *C*. *jejuni* strains is considered a serious public health problem [27]. A European Union summary report has shown that many worldwide studies have reported on high levels of resistance to ciprofloxacin, nalidixic acid, and tetracycline [28]. Furthermore, a worrying emerging resistance to macrolides was recently observed for *Campylobacter* [16, 28–29]. In Europe, the rates of fluoroquinolones-resistance in broilers are highly variable, ranging from 1.2% in Norway [30] to 44% in Belgium [31]. An alarming situation was found in Spain where the resistance rate to fluoroquinolones was reported to be 90% [32], while in Poland ciprofloxacin resistance increased from 47.9% during 2005–2008 to 90.2% during 2005–2008 [29]. Several studies from Denmark and Finland have reported that fluoroquinolones and tetracycline resistance rates were significantly higher in travel-associated infections when compared with domestically acquired infections [33–34]. Interestingly, it appears that fluoroquinolone resistance has emerged on poultry farms even in the absence of the above-mentioned antimicrobials [35]. It has been also been suggested that other antimicrobials may select for fluoroquinolone-resistance in *Campylobacter* [36], but the mechanisms involved are still not completely clarified. For these reasons, continuous monitoring of the resistance rates and investigating the resistance mechanisms is fundamental to combating the potential spread of AMR *C*. *jejuni* in humans, as well as across the food chain and in the environment. The present study was undertaken to provide better insight into the dynamics of antibiotic resistance in *C*. *jejuni* in Italy by characterising *C*. *jejuni* strains from humans, poultry and birds from wild and urban habitats. We also sought to determine whether a correlation exists between the resistance phenotypes and genotypes of the *C*.*jejuni* isolates from this study.

Our results show that ciprofloxacin and nalidixic acid resistance was very high in the isolates from humans and poultry (range: 74% to 85%), while 67% of the poultry isolates and approximately half of the human isolates displayed tetracycline resistance. These levels of resistance are consistent with those reported by other recent studies [37]. Similar findings were observed in our previous study of AMR in *C*. *jejuni* isolated from broilers, where we noted higher rates of resistance against fluoroquinolones (90%) and similar rates of resistance against tetracycline (64%) [38].

Fluoroquinolones and tetracycline have been used to treat infections in poultry and as growth promoters over the last 50 years [39]. Hence, the high resistance rates to these antimicrobials are likely to be the consequence of their continuous over use [40]. Consistent with this, we observed much lower levels of antibiotic resistance in the isolates obtained from the birds living in urban and wild habitats, which reinforces the argument that the extreme levels of AMR observed in the strains from poultry result from the common use of antibiotics in the farm environment. The MDR profiles of birds from the urban and non-urban habitats suggest that both types of birds could be an important reservoir of MDR *C*.*jejuni* strains, and a potential risk population for the spread of resistant bacteria.

Low levels of gentamycin, streptomycin and erythromycin resistance were observed in this study, a finding concordant with previous studies reporting low resistance levels to these antimicrobials in *C*. *jejuni* isolated from broiler meat [37–39, 41]. In the present study, 7.84% of the human isolates showed resistance to erythromycin, a higher rate than that identified in similar strains by other authors [42–45] but similar to that reported in a recent study [46]. A recent European Union report stated that the mean European level of erythromycin resistance in *C*. *jejuni* was 2.1% in the 21,993 tested isolates [28]. However, a worrying increase in intermediate susceptibility to erythromycin (82.09%) was observed for birds from wild and urban habitats.

MDR, which is defined as resistance to three or more antimicrobial classes [47], has greatly increased worldwide in *C*. *jejuni* [39]. In the present study, 47.90% of the chicken strains and 17.76% of the human strains showed MDR phenotypes towards fluoroquinolones and tetracycline, demonstrating the severity of the problem linked to increases in AMR in microorganisms. Although sampling from the birds in the urban habitat was limited, we observed worrying MDR profiles in them, reaching up to five and six drugs. Nevertheless, the infection prevalence rates were statistically significant in the urban habitat-associated birds, suggesting their potential as a vehicle for the transmission of pathogenic *C*. *jejuni* and AMR traits to humans.

We screened the quinolone-resistant *C*. *jejuni* isolates for the presence of mutations in the QRDR of the *gyr*A gene. The T86I amino acid substitution was found to be the most common; indeed, it was present in 97.43% and 96.22% of the isolates from humans and poultry, respectively, displaying resistance to ciprofloxacin and nalidixic acid. However, isolates from birds inhabiting the wild and urban study areas harboured a lower percentage of this mutation (44.44%).

We identified another amino acid substitution, T86V, in only eight chicken isolates. Nine poultry isolates were resistant to ciprofloxacin, but they lacked this mutation. These results seem to confirm that quinolone resistance does not depend exclusively on the aforementioned mutations, but can also be attributed to other and/or unknown resistance mechanisms, such as the efflux pump system, as has been also reported in other studies [48–50].

The CmeABC multidrug efflux system, which is the best described multidrug efflux pump to date, plays an important role in antimicrobial resistance. It was present in all the isolates we analysed. The efflux system consists of an outer membrane protein (encoded by *CmeC*), an internal membrane drug transporter (encoded by *CmeB*) and a periplasmic protein (encoded by *CmeA*). Together, these components form a membrane channel that expels toxic substances from the cell [49]. In *C*. *jejuni*, the *cmeABC* operon is negatively regulated by the cmeR repressor, which binds to a 16-base inverted repeat sequence (called the *cmeR-*Box) located in the promoter region of the *cmeABC* operon [50–52]. Because a single-nucleotide insertion or deletion in the *cmeR*-Box has been shown to lead to reduced binding by *CmeR*, this might in turn increase the expression of *cmeABC* and enhance the ciprofloxacin resistance level in *C*. *jejuni* isolates [53].

The *blaOXA-61* gene has been shown to confer resistance to beta-lactams in *C*. *jejuni* strains [54]. Over 70% of the human isolates and 80% of the isolates from the wild non-urban habitat birds possessed *blaOXA-61 and blaOXA-184* genes, respectively, a finding reported by other authors also [54–55]. However, *Campylobacter* is intrinsically resistant to beta-lactams; therefore, this class of molecules is not recommended for treating infections caused by this bacterium.

We noted a high correlation between phenotypic resistance to tetracycline and quinolones and the presence of one or more resistance genes or the nucleotide polymorphisms expected to confer resistance to these antimicrobials. For tetracycline, the correlation varied between 88% and 93.27% for the presence of a putative resistance gene and the observed resistance phenotype. A few discrepancies were found with respect to the *gyr*A mutation and the observed phenotype for the isolates from the birds from wild habitats, which may be explained by the existence of efflux pump mechanisms.

## Conclusions

The results of our study suggest that antimicrobial resistance in *C*. *jejuni* isolated from humans is correlated with the use of antibiotics in veterinary medicine, and that antimicrobial over use/inappropriate use is an important selective process. Our findings also suggest that multiple resistance patterns to several classes of antibiotics continue to emerge in *C*. *jejuni*. Considering the genomic plasticity of *Campylobacter* and its commensalism with various animal species that are likely to be exposed to different antibiotics, additional resistance mechanisms may continue to evolve in this bacterium. Our data clearly show that antibiotic resistance in *Campylobacter* is rising. Therefore, AMR monitoring is crucial for designing containment strategies for zoonotic microorganisms like *Campylobacter* because proper monitoring should help to foresee future AMR spread in animal populations, in humans, and in environmental bacterial populations.

As macrolides are the current treatment choice for campylobacteriosis, the emergence of widespread macrolide resistance remains of primary interest. Knowledge about which genetic resistance elements are present in a bacterial population is crucial for the successful development of new programs of foodborne disease surveillance and control. Phenotypic susceptibility testing, in our opinion, remains fundamental to being able to detect resistance to the principal antimicrobials, even when traditional antibiotic panels can only test a limited number of antibiotics. Additional molecular approaches, such as genomics or proteomics are therefore required to provide new insights into the molecular mechanisms involved in the development of antibiotic resistance in *Campylobacter*.

To provide information for agricultural production systems and the associated veterinary usage of antimicrobial pharmaceuticals, we also assessed AMR in birds from wild and urban habitats. By addressing the linkage between livestock and wildlife, our study has provided preliminary insight into the potential role of wildlife to act as vectors, reservoirs or amplifiers of antimicrobial resistant microbes. We suggest using birds from wild and urban habitats as key sentinel animals for the surveillance of ecosystem contamination.

## Supporting information

S1 Table Frequency of antimicrobial resistance genes (*tet* (O), *cmeA*, *cmeB*, *cmeC*, *cmeR*, *blaOXA-61* and *blaOXA-184*) and *gyrA*-associated point mutations in *C*.*jejuni* from poultry and bird isolates. (PDF)
