# Correction: Antimicrobial resistance genotypes and phenotypes of *Campylobacter jejuni* isolated in Italy from humans, birds from wild and urban habitats, and poultry

**DOI:** 10.1371/journal.pone.0225231

**Published:** 2019-11-07

**Authors:** Francesca Marotta, Giuliano Garofolo, Lisa Di Marcantonio, Gabriella Di Serafino, Diana Neri, Romina Romantini, Lorena Sacchini, Alessandra Alessiani, Guido Di Donato, Roberta Nuvoloni, Anna Janowicz, Elisabetta Di Giannatale

The third author's name is spelled incorrectly. The correct name is: Lisa Di Marcantonio. The correct citation is: Marotta F, Garofolo G, Di Marcantonio L, Di Serafino G, Neri D, Romantini R, et al. (2019) Antimicrobial resistance genotypes and phenotypes of *Campylobacter jejuni* isolated in Italy from humans, birds from wild and urban habitats, and poultry. PLoS ONE 14(10): e0223804. https://doi.org/10.1371/journal.pone.0223804.
