# Supplementary material for: Antimicrobial resistance genotypes and phenotypes of Campylobacter jejuni isolated in Italy from humans, birds from wild and urban habitats, and poultry
Source: PLoS One. 2019 Oct 11;14(10):e0223804. doi: 10.1371/journal.pone.0223804 (PMC6788699; doi:10.1371/journal.pone.0223804)
Supplement: S1 Table — (PDF) [file pone.0223804.s001.pdf]

Assembly ID	Source	TAG	GyrA_AA_86	OXA-184	OXA-61	cmeA	cmeB	cmeC	cmeR	tetO
2015-TE-10529.fasta	Chicken food	Chicken	I	100	.	100	99.9	100	100	100
2015-TE-10534.fasta	Chicken animals	Chicken	I	100	.	100	99.9	100	100	100
2015-TE-10538.fasta	Chicken food	Chicken	I	100	.	100	99.9	100	100	100
2015-TE-10544.fasta	Chicken food	Chicken	I	.	.	100	99.9	100	100	99.9
2015-TE-10545.fasta	Chicken animals	Chicken	I	.	.	100	99.9	100	100	99.9
2015-TE-10553.fasta	Chicken food	Chicken	I	.	.	100	99.9	100	100	99.9
2015-TE-11797.fasta	Chicken animals	Chicken	I	.	100	100	99,9	100	100	100
2015-TE-11801.fasta	Chicken animals	Chicken	I	100	.	100	99.9	100	100	.
2015-TE-11813.fasta	Chicken animals	Chicken	I	.	100	100	100	100	100	99.9
2015-TE-11820.fasta	Chicken animals	Chicken	I	.	100	100	100	100	100	99.9
2015-TE-11821.fasta	Chicken animals	Chicken	I	100	.	100	99.9	100	100	100
2015-TE-11831.fasta	Chicken animals	Chicken	I	.	100	100	100	100	100	100
2015-TE-11833.fasta	Chicken animals	Chicken	I	.	100	100	99.9	100	100	100
2015-TE-11834.fasta	Chicken animals	Chicken	I	.	100	100	99.9	100	100	100
2015-TE-11835.fasta	Chicken animals	Chicken	I	.	100	100	99.9	100	100	100
2015-TE-11836.fasta	Chicken food	Chicken	I	.	100	100	99.9	100	100	100
2015-TE-12208.fasta	Chicken animals	Chicken	T	.	100	100	100	100	100	.
2015-TE-12214.fasta	Chicken animals	Chicken	T	.	100	100	100	100	100	.
2015-TE-12215.fasta	Chicken food	Chicken	T	.	100	100	100	100	100	.
2015-TE-12221.fasta	Chicken food	Chicken	T	.	100	100	100	100	100	.
2015-TE-12222.fasta	Chicken food	Chicken	T	.	100	100	100	100	100	.
2015-TE-12229.fasta	Chicken animals	Chicken	T	.	100	100	100	100	100	.
2015-TE-12232.fasta	Chicken food	Chicken	T	.	100	100	100	100	100	.
2015-TE-12237.fasta	Chicken food	Chicken	I	.	.	99.91	99.55	99.93	100	.
2015-TE-12793.fasta	Chicken animals	Chicken	I	100	.	100	99.9	100	100	100
2015-TE-12794.fasta	Chicken animals	Chicken	I	100	.	99.91	99.55	100	100	100
2015-TE-13054.fasta	Chicken animals	Chicken	I	100	.	100	99.9	100	100	100
2015-TE-13056.fasta	Chicken animals	Chicken	I	100	.	100	99.9	100	100	100
2015-TE-13057.fasta	Chicken animals	Chicken	I	100	.	100	99.9	100	100	100
2015-TE-13525.fasta	Chicken food	Chicken	T	.	100	100	99.55	100	100	100
2015-TE-13529.fasta	Chicken animals	Chicken	I	.	100	100	100	100	100	99.9

2015-TE-13534.fasta	Chicken food	Chicken	I	.	100	100	100	100	100	99.9
2015-TE-13538.fasta	Chicken food	Chicken	T	.	100	100	99.9	100	99.84	.
2015-TE-13539.fasta	Chicken animals	Chicken	T	.	100	100	99.9	100	99.84	.
2015-TE-13540.fasta	Chicken animals	Chicken	T	88.62	.	100	99.9	100	100	.
2015-TE-13544.fasta	Chicken food	Chicken	T	88.62	.	100	99.9	100	100	.
2015-TE-14308.fasta	human	Human	T	.	100	100	100	100	100	.
2015-TE-14447.fasta	Chicken animals	Chicken	I	.	100	100	100	100	100	99.9
2015-TE-14491-1-1.fasta	whitewagtail	Wild_bird	I	.	100	100	100	100	100	99.9
2015-TE-14493-1-1.fasta	starling	Urban_bird	T	.	100	100	100	100	100	.
2015-TE-14511.fasta	Chicken animals	Chicken	I	.	100	100	100	100	100	99,9
2015-TE-14523.fasta	Chicken animals	Chicken	I	100	.	100	99.9	100	100	100
2015-TE-14532.fasta	Chicken animals	Chicken	I	.	100	100	99.9	100	100	100
2015-TE-14618.fasta	Chicken animals	Chicken	I	.	100	100	100	100	100	99,9
2015-TE-14675.fasta	Chicken animals	Chicken	I	.	100	100	100	100	100	99,9
2015-TE-14798.fasta	Chicken animals	Chicken	I	.	100	100	100	100	100	99.9
2015-TE-14806.fasta	Chicken food	Chicken	I	.	100	100	100	100	100	99.9
2015-TE-14855.fasta	Chicken food	Chicken	I	.	100	100	100	100	100	.
2015-TE-14856.fasta	Chicken animals	Chicken	I	.	100	100	100	100	100	.
2015-TE-14865.fasta	Chicken animals	Chicken	I	.	100	100	100	100	100	99.9
2015-TE-14871.fasta	Chicken food	Chicken	I	.	100	100	100	100	100	99.9
2015-TE-15342.fasta	Chicken animals	Chicken	I	100	.	100	99.9	100	100	100
2015-TE-15346.fasta	Chicken food	Chicken	I	100	.	100	99.9	99.93	100	79.74
2015-TE-15582.fasta	Chicken animals	Chicken	I	.	100	100	100	100	100	99.9
2015-TE-15584.fasta	Chicken animals	Chicken	I	100	.	100	99.9	100	100	100
2015-TE-15588.fasta	Chicken animals	Chicken	I	99.6	.	100	99.9	100	100	.
2015-TE-15873.fasta	Chicken animals	Chicken	T	.	100	100	100	100	100	.
2015-TE-15879.fasta	Chicken animals	Chicken	I	.	100	100	100	100	100	99.9
2015-TE-15930.fasta	Chicken animals	Chicken	I	.	100	100	100	100	100	99,9
2015-TE-15934.fasta	Chicken animals	Chicken	I	.	100	100	100	100	100	99.9
2015-TE-15958.fasta	Chicken animals	Chicken	I	.	100	100	100	100	100	99.9
2015-TE-15988.fasta	Chicken animals	Chicken	I	100	.	100	99.9	100	100	100
2015-TE-16019.fasta	Chicken food	Chicken	T	.	100	100	100	100	100	.

2015-TE-16020.fasta	Chicken animals	Chicken	I	.	100	100	100	100	100	99.9
2015-TE-16033.fasta	Chicken animals	Chicken	I	100	.	100	100	100	100	.
2015-TE-16035.fasta	Chicken food	Chicken	I	.	100	100	100	100	100	99.9
2015-TE-16051.fasta	Chicken food	Chicken	I	.	100	100	100	100	100	.
2015-TE-17432-1-1.fasta	magpie	Urban_bird	T	.	100	100	99.9	100	100	.
2015-TE-17433-1-1.fasta	magpie	Urban_bird	T	88.62	.	100	100	100	100	.
2015-TE-17434-1-1.fasta	magpie	Urban_bird	T	.	100	100	99.9	100	100	.
2015-TE-17435-1-1.fasta	magpie	Urban_bird	T	.	100	100	99.9	100	100	.
2015-TE-17436-1-1.fasta	magpie	Urban_bird	T	.	100	100	99.9	100	100	.
2015-TE-17622.fasta	human	Human	I	.	100	100	100	100	100	99.9
2015-TE-17888.fasta	Chicken animals	Chicken	I	.	100	99.91	99.55	99.93	100	100
2015-TE-17890.fasta	Chicken animals	Chicken	I	.	100	100	100	100	100	99.9
2015-TE-17891.fasta	Chicken animals	Chicken	I	.	100	99.91	99.55	99.93	100	100
2015-TE-18078.fasta	Chicken animals	Chicken	I	.	100	100	100	100	100	99.9
2015-TE-18079.fasta	Chicken animals	Chicken	I	.	100	100	100	100	100	99.9
2015-TE-18082.fasta	Chicken animals	Chicken	I	100	.	100	99.9	100	100	100
2015-TE-18084.fasta	Chicken animals	Chicken	I	100	.	100	99.9	100	100	100
2015-TE-18086.fasta	Chicken animals	Chicken	I	.	100	100	100	100	100	99.9
2015-TE-18089.fasta	Chicken animals	Chicken	T	.	100	100	100	100	100	.
2015-TE-18090.fasta	Chicken animals	Chicken	I	100	.	100	99.9	100	100	100
2015-TE-18091.fasta	Chicken animals	Chicken	I	100	.	100	99.9	100	100	100
2015-TE-18099.fasta	Chicken animals	Chicken	V	.	100	100	100	100	100	100
2015-TE-18178.fasta	Chicken animals	Chicken	I	99.6	.	100	99.9	100	100	23.23
2015-TE-18180.fasta	Chicken animals	Chicken	I	99.6	.	100	99.9	100	100	.
2015-TE-18181.fasta	Chicken animals	Chicken	I	99.6	.	100	99.9	100	100	.
2015-TE-18182.fasta	Chicken animals	Chicken	I	99.6	.	100	99.9	100	100	.
2015-TE-18184.fasta	Chicken animals	Chicken	I	.	100	100	100	100	100	99.9
2015-TE-18185.fasta	Chicken animals	Chicken	I	.	100	100	100	100	100	99.9
2015-TE-18188.fasta	Chicken animals	Chicken	I	.	100	100	100	100	100	100
2015-TE-18193.fasta	Chicken animals	Chicken	I	.	100	100	100	100	100	99.9
2015-TE-18195.fasta	Chicken animals	Chicken	I	100	.	99.91	99.55	100	100	100
2015-TE-18196.fasta	Chicken animals	Chicken	I	100	.	99.91	99.55	100	100	100

2015-TE-18263.fasta	Chicken animals	Chicken	I	.	.	100	99.9	100	100	99.9
2015-TE-18264.fasta	Chicken animals	Chicken	I	.	.	100	99.9	100	100	99.9
2015-TE-18290.fasta	Chicken animals	Chicken	I	.	100	100	99.9	100	100	99.9
2015-TE-18298.fasta	Chicken animals	Chicken	I	.	100	100	99.9	100	100	99.9
2015-TE-18300.fasta	Chicken animals	Chicken	T	.	100	100	100	100	100	100
2015-TE-18304.fasta	Chicken animals	Chicken	T	.	100	100	100	100	100	100
2015-TE-18310.fasta	Chicken animals	Chicken	T	.	100	100	99.9	100	100	.
2015-TE-18724.fasta	Chicken animals	Chicken	I	100	.	100	99.9	100	100	100
2015-TE-18869-1-1.fasta	Crow	Urban_bird	T	88.62	.	100	100	100	100	.
2015-TE-18871-1-1.fasta	Crow	Urban_bird	T	88.62	.	100	100	100	100	.
2015-TE-19309-1-1.fasta	Chicken animals	Chicken	I	.	.	100	99,9	100	100	99.9
2015-TE-19316.fasta	Chicken animals	Chicken	I	.	100	100	99.9	100	100	99.9
2015-TE-19322.fasta	Chicken food	Chicken	I	.	.	100	99,9	100	100	99.9
2015-TE-19324.fasta	Chicken animals	Chicken	I	100	.	99.91	99.55	99.93	100	100
2015-TE-19326.fasta	Chicken food	Chicken	I	100	.	99.91	99.55	99.93	100	100
2015-TE-19331.fasta	Chicken food	Chicken	I	.	.	99.91	99.55	99.93	100	.
2015-TE-19334.fasta	Chicken food	Chicken	I	.	.	100	99.9	100	100	99.9
2015-TE-19340.fasta	Chicken animals	Chicken	I	.	100	100	100	100	100	99.9
2015-TE-19477.fasta	Chicken food	Chicken	I	.	100	100	99.9	100	100	100
2015-TE-19480.fasta	Chicken animals	Chicken	I	.	100	100	100	100	100	99.9
2015-TE-19482.fasta	Chicken food	Chicken	I	100	.	99.91	99.55	100	100	100
2015-TE-19486.fasta	Chicken food	Chicken	I	.	100	100	100	100	100	99.9
2015-TE-19487.fasta	Chicken animals	Chicken	I	.	100	100	100	100	100	99.9
2015-TE-19488.fasta	Chicken animals	Chicken	I	.	100	100	100	100	100	99.9
2015-TE-19489.fasta	Chicken animals	Chicken	I	.	100	100	100	100	100	99.9
2015-TE-19491.fasta	Chicken animals	Chicken	I	.	100	100	100	100	100	99.9
2015-TE-19492.fasta	Chicken food	Chicken	I	.	100	100	100	100	100	99.9
2015-TE-19494.fasta	Chicken food	Chicken	I	.	100	100	99.9	100	100	100
2015-TE-19498.fasta	Chicken food	Chicken	I	.	100	99.91	99.55	99.93	100	100
2015-TE-19788.fasta	Chicken animals	Chicken	I	.	.	100	99.9	100	100	99.9
2015-TE-19794.fasta	Chicken animals	Chicken	T	.	100	100	99.9	100	100	.
2015-TE-19885.fasta	Chicken animals	Chicken	I	.	100	100	100	100	100	99.9

2015-TE-19904.fasta	Chicken animals	Chicken	I	100	.	100	100	100	100	99.9
2015-TE-19909.fasta	Chicken animals	Chicken	I	100	.	100	100	100	100	99.9
2015-TE-19962.fasta	Chicken animals	Chicken	I	100	.	100	99.9	100	100	100
2015-TE-19973.fasta	Chicken animals	Chicken	I	100	.	99.91	99.55	99.93	100	100
2015-TE-19991.fasta	Chicken animals	Chicken	T	.	100	100	100	100	100	100
2015-TE-20117.fasta	Chicken food	Chicken	I	.	100	100	100	100	100	99,9
2015-TE-20118.fasta	Chicken food	Chicken	I	.	100	100	100	100	100	99.9
2015-TE-20121.fasta	Chicken food	Chicken	I	.	100	100	100	100	100	99.9
2015-TE-20126-1-1.fasta	owl	Wild_bird	T	97.19	.	100	99.9	100	100	.
2015-TE-20389.fasta	human	Human	I	.	100	100	99.9	100	100	.
2015-TE-20519-1-1.fasta	Chicken food	Chicken	I	.	.	100	99.9	100	100	99.9
2015-TE-20594.fasta	human	Human	I	.	.	100	99.9	100	100	99.9
2015-TE-20596.fasta	human	Human	T	.	100	100	100	100	100	.
2015-TE-20598.fasta	human	Human	I	.	100	100	100	100	100	99.9
2015-TE-20602.fasta	human	Human	I	97.32	.	100	99.9	100	100	.
2015-TE-20607.fasta	human	Human	I	.	100	100	100	100	100	100
2015-TE-20614.fasta	human	Human	T	.	.	100	99.9	100	100	100
2015-TE-20668.fasta	Chicken animals	Chicken	I	.	.	100	99.9	100	100	99.9
2015-TE-20672.fasta	Chicken food	Chicken	I	.	100	100	100	100	100	99.9
2015-TE-20674-1-1.fasta	Chicken animals	Chicken	I	.	100	100	100	100	100	99.9
2015-TE-20679.fasta	Chicken food	Chicken	I	100	.	99.91	99.55	99.93	100	100
2015-TE-20680.fasta	Chicken animals	Chicken	I	100	.	99.91	99.55	99.93	100	100
2015-TE-20685.fasta	Chicken animals	Chicken	I	.	100	100	100	100	100	.
2015-TE-20691.fasta	Chicken animals	Chicken	V	.	7.11;100.00	16.65;88.05;4.98;6.24	100	100	100	100
2015-TE-20692.fasta	Chicken animals	Chicken	T	.	100	100	100	100	100	.
2015-TE-20693.fasta	Chicken animals	Chicken	I	.	100	100	99.9	100	100	99.9
2015-TE-20696.fasta	Chicken animals	Chicken	I	100	.	99.91	99.55	100	100	100
2015-TE-20698.fasta	Chicken animals	Chicken	I	.	100	100	100	100	100	100
2015-TE-20702.fasta	Chicken animals	Chicken	I	.	.	100	99.9	100	100	99,9
2015-TE-20703.fasta	Chicken animals	Chicken	I	100	.	99.91	99.55	100	100	100
2015-TE-20704.fasta	Chicken animals	Chicken	I	100	.	99.91	99.55	100	100	100
2015-TE-20706.fasta	Chicken animals	Chicken	I	.	100	100	99.9	100	100	99.9

2015-TE-20707.fasta	Chicken animals	Chicken	I	100	.	99.91	99.55	99.93	100	39.38;45.99;20.26
2015-TE-20708.fasta	Chicken animals	Chicken	I	.	100	100	100	100	100	100
2015-TE-20713.fasta	Chicken animals	Chicken	I	100	.	100	99.9	100	100	99.9
2015-TE-20716.fasta	Chicken food	Chicken	I	100	.	100	99.9	100	100	99.9
2015-TE-20721.fasta	Chicken food	Chicken	I	100	.	100	99.9	100	100	100
2015-TE-20723.fasta	Chicken animals	Chicken	V	.	100	100	100	100	100	100
2015-TE-20725.fasta	Chicken animals	Chicken	I	.	100	100	100	100	100	.
2015-TE-20726-1-1.fasta	Chicken food	Chicken	I	.	100	100	100	100	100	.
2015-TE-20733.fasta	Chicken food	Chicken	I	100	.	99.91	99.55	99.93	100	100
2015-TE-20744.fasta	Chicken food	Chicken	I	.	100	100	100	100	100	99.90;99.90
2015-TE-20756.fasta	Chicken animals	Chicken	I	.	100	100	100	100	100	99.9
2015-TE-20764.fasta	Chicken food	Chicken	T	88.62	.	100	99.9	100	100	.
2015-TE-20770.fasta	Chicken animals	Chicken	I	.	.	100	99.9	100	100	99.9
2015-TE-20774.fasta	Chicken food	Chicken	I	.	.	100	99.9	100	100	99.9
2015-TE-21506.fasta	Chicken animals	Chicken	V	.	100	100	100	100	100	100
2015-TE-22020.fasta	Chicken animals	Chicken	I	100	.	99.91	99.55	99.93	100	39.38;45.99;20.26
2015-TE-22656.fasta	Chicken animals	Chicken	I	.	100	100	100	100	100	99.9
2015-TE-22940.fasta	Chicken animals	Chicken	I	.	100	100	100	100	100	99.9
2015-TE-22942.fasta	Chicken food	Chicken	I	.	100	100	100	100	100	99.9
2015-TE-22943.fasta	Chicken food	Chicken	I	100	.	100	99.9	100	100	100
2015-TE-22953.fasta	Chicken animals	Chicken	I	.	100	100	99.9	100	100	99.9
2015-TE-22955.fasta	Chicken food	Chicken	I	.	100	100	99.9	100	100	99.9
2015-TE-22961.fasta	Chicken animals	Chicken	I	.	100	99.91	99.55	99.93	100	100
2015-TE-22962.fasta	Chicken food	Chicken	I	.	100	99.91	99.55	99.93	100	100
2015-TE-23067.fasta	Chicken animals	Chicken	I	100	.	99.91	99.55	99.93	100	100
2015-TE-23071.fasta	Chicken animals	Chicken	I	.	.	100	99.9	100	100	99.9
2015-TE-23074.fasta	Chicken animals	Chicken	T	.	.	100	100	100	100	.
2015-TE-23773.fasta	Chicken animals	Chicken	I	.	100	99.91	99.55	99.93	100	100
2015-TE-23774.fasta	Chicken food	Chicken	I	.	100	99.91	99.55	99.93	100	100
2015-TE-23780.fasta	Chicken animals	Chicken	I	.	100	100	100	100	100	.
2015-TE-23789-1-1.fasta	Chicken animals	Chicken	I	100	.	99.91	99.55	99.93	100	100
2015-TE-23790.fasta	Chicken food	Chicken	I	100	.	99.91	99.55	99.93	100	100

2015-TE-23857.fasta	Chicken animals	Chicken	I	.	100	100	99.9	100	100	100
2015-TE-23858.fasta	Chicken food	Chicken	T	100	.	100	100	100	100	.
2015-TE-23865.fasta	Chicken animals	Chicken	I	.	100	100	99.9	100	100	100
2015-TE-23866.fasta	Chicken food	Chicken	T	.	100	100	100	100	100	.
2015-TE-23874.fasta	Chicken food	Chicken	I	100	.	99.91	99.55	100	100	100
2015-TE-23876.fasta	Chicken food	Chicken	I	100	.	100	99.9	100	100	100
2015-TE-23885.fasta	Chicken food	Chicken	I	.	100	100	99.9	100	100	99.9
2015-TE-23896.fasta	Chicken food	Chicken	I	.	100	100	100	100	100	99.9
2015-TE-23897.fasta	Chicken food	Chicken	I	100	.	100	99.9	100	100	99.9
2015-TE-23930-1-1.fasta	Chicken food	Chicken	I	.	100	100	100	100	100	99.90;11.41
2015-TE-23983-1-1.fasta	greenfinch	Wild_bird	T	.	100	100	99.9	100	100	.
2015-TE-23984-1-1.fasta	greenfinch	Wild_bird	T	.	100	100	99.9	100	100	.
2015-TE-24014.fasta	Chicken animals	Chicken	I	100	.	100	99.9	100	100	100
2015-TE-24033.fasta	Chicken animals	Chicken	I	.	100	99.91	99.55	100	100	100
2015-TE-24037.fasta	Chicken animals	Chicken	I	.	100	99.91	99.55	100	100	100
2015-TE-24038.fasta	Chicken animals	Chicken	I	.	100	100	100	100	100	.
2015-TE-24292.fasta	Chicken animals	Chicken	I	100	.	99.91	99.55	99.93	100	99,9
2015-TE-24308.fasta	Chicken animals	Chicken	I	100	.	100	99.9	100	100	100
2015-TE-24309.fasta	Chicken food	Chicken	I	100	.	100	99.9	100	100	100
2015-TE-24329.fasta	Chicken animals	Chicken	I	100	.	100	99.9	99.93	100	100
2015-TE-24330.fasta	Chicken animals	Chicken	I	.	100	100	100	100	100	.
2015-TE-24332.fasta	Chicken food	Chicken	I	.	100	100	100	100	100	99.9
2015-TE-24333.fasta	Chicken animals	Chicken	I	.	100	100	100	100	100	99.9
2015-TE-24335.fasta	Chicken animals	Chicken	I	.	100	100	100	100	100	99.9
2015-TE-24342.fasta	Chicken animals	Chicken	T	.	99.87	100	100	100	100	100
2015-TE-24344.fasta	Chicken food	Chicken	I	.	100	100	100	100	100	.
2015-TE-24350.fasta	Chicken animals	Chicken	T	.	99.87	100	100	100	100	100
2015-TE-24352.fasta	Chicken food	Chicken	T	.	99.87	100	100	100	100	100
2015-TE-24454.fasta	Chicken animals	Chicken	T	.	100	100	99.9	100	100	17.81;25.73
2015-TE-25085.fasta	Chicken animals	Chicken	I	100	.	100	99.9	100	100	99.9
2015-TE-25086.fasta	Chicken animals	Chicken	I	100	.	100	99.9	100	100	99.9
2015-TE-25087.fasta	Chicken animals	Chicken	I	.	100	100	100	100	100	99.9

2015-TE-25089.fasta	Chicken animals	Chicken	I	.	100	100	100	100	100	99.9
2015-TE-25090.fasta	Chicken animals	Chicken	T	.	100	100	99.9	100	100	.
2015-TE-25795.fasta	Chicken animals	Chicken	I	.	100	100	100	100	100	.
2015-TE-25797.fasta	Chicken animals	Chicken	I	.	100	100	100	100	100	99.9
2015-TE-25798.fasta	Chicken animals	Chicken	T	.	100	100	99.9	100	100	.
2015-TE-25799.fasta	Chicken animals	Chicken	I	100	.	100	99.9	100	100	100
2015-TE-25804.fasta	Chicken animals	Chicken	I	.	.	100	100	100	100	.
2015-TE-25809.fasta	Chicken animals	Chicken	I	.	100	100	100	100	100	99.9
2015-TE-26013.fasta	Chicken animals	Chicken	I	100	.	99.91	99.55	99.93	100	100
2015-TE-26646-1-1.fasta	Chicken food	Chicken	I	.	100	100	100	100	100	99.9
2015-TE-26647-1-1.fasta	Chicken food	Chicken	T	.	100	100	100	100	100	.
2015-TE-26909.fasta	Chicken animals	Chicken	I	100	.	100	99.9	99,93	100	100
2015-TE-26910.fasta	Chicken food	Chicken	I	100	.	100	99.9	99.93	100	100
2015-TE-26928.fasta	Chicken animals	Chicken	I	100	.	100	99.9	99.93	100	100
2015-TE-26930.fasta	Chicken animals	Chicken	I	.	100	100	100	100	100	99.9
2015-TE-26934.fasta	Chicken animals	Chicken	I	.	.	100	100	100	100	.
2015-TE-27015.fasta	Chicken food	Chicken	I	100	.	100	99.9	100	100	100
2015-TE-27162.fasta	Chicken animals	Chicken	I	.	100	100	100	100	100	99.9
2015-TE-27165.fasta	Chicken animals	Chicken	I	100	.	100	100	100	100	.
2015-TE-27202.fasta	Chicken animals	Chicken	I	.	100	100	100	100	100	99.9
2015-TE-27206.fasta	Chicken animals	Chicken	I	.	100	100	100	100	100	99.9
2015-TE-27207.fasta	Chicken animals	Chicken	I	.	100	100	100	41.65;16.29;16.29;16.29;74.65	100	99.9
2015-TE-27218.fasta	Chicken animals	Chicken	I	100	.	99.91	99.55	99.93	100	100
2015-TE-27228.fasta	Chicken animals	Chicken	I	.	100	100	100	100	100	99,9
2015-TE-27230.fasta	Chicken animals	Chicken	I	.	100	100	100	100	100	99.9
2015-TE-27231.fasta	Chicken animals	Chicken	I	.	100	100	100	100	100	99.9
2015-TE-27232-1-1.fasta	Chicken food	Chicken	I	.	100	100	100	100	100	99.9
2015-TE-27233.fasta	Chicken animals	Chicken	I	.	100	100	100	100	100	99.9
2015-TE-27236-1-1.fasta	Chicken food	Chicken	T	.	100	100	100	100	100	.
2015-TE-27237.fasta	Chicken animals	Chicken	I	.	100	100	100	100	100	.
2015-TE-27553-1-1.fasta	Chicken food	Chicken	I	.	100	100	100	100	100	99.9
2015-TE-27554-1-1.fasta	Chicken food	Chicken	I	100	.	100	99.9	100	100	99.9

2015-TE-27556-1-1.fasta	Chicken food	Chicken	T	.	93.54	100	99.9	99.93	100	.
2015-TE-27604.fasta	Chicken animals	Chicken	I	.	100	100	100	100	100	.
2015-TE-27605.fasta	Chicken animals	Chicken	I	100	.	99.91	99.55	99.93	100	100
2015-TE-27608.fasta	Chicken animals	Chicken	T	.	100	100	99.9	100	99.84	.
2015-TE-27611.fasta	Chicken animals	Chicken	T	.	100	100	99.9	100	99.84	.
2015-TE-27612.fasta	Chicken animals	Chicken	I	100	.	100	100	100	100	.
2015-TE-27626.fasta	Chicken food	Chicken	T	.	100	100	100	100	100	.
2015-TE-27628.fasta	Chicken food	Chicken	I	100	.	99.91	99.55	100	100	100
2015-TE-27635.fasta	Chicken food	Chicken	T	.	100	100	100	100	100	.
2015-TE-27642.fasta	Chicken animals	Chicken	T	.	93.54	100	99.9	99.93	100	.
2015-TE-27644.fasta	Chicken food	Chicken	T	.	93.54	100	99.9	99.93	100	.
2015-TE-28098.fasta	Chicken food	Chicken	T	.	93.54	100	99.9	99.93	100	.
2015-TE-28133.fasta	Chicken food	Chicken	T	.	93.54	100	99,9	99.93	100	.
2015-TE-28139.fasta	Chicken food	Chicken	I	.	100	100	100	100	100	100
2015-TE-28140.fasta	Chicken animals	Chicken	I	.	100	100	100	100	100	100
2015-TE-28147.fasta	Chicken food	Chicken	T	97.19	.	100	99.9	100	100	.
2015-TE-28179-1-1.fasta	Chicken food	Chicken	I	100	.	99.91	99.55	100	100	100
2015-TE-28365-1-1.fasta	pigeons	Urban_bird	T	99.6	.	100	99.9	100	100	.
2015-TE-28416.fasta	Chicken animals	Chicken	I	100	.	99.91	99.55	99.93	100	100
2015-TE-28417.fasta	Chicken animals	Chicken	I	.	100	100	100	100	100	99.9
2015-TE-28882-1-1.fasta	Chicken food	Chicken	I	.	.	100	99.9	100	100	99.9
2015-TE-28884-1-1.fasta	Chicken food	Chicken	I	100	.	99.91	99.55	99.93	100	100
2015-TE-28885-1-1.fasta	Chicken food	Chicken	I	.	100	100	99.9	100	100	100
2015-TE-29065-1-1.fasta	Chicken food	Chicken	I	100	.	99.91	99.55	100	100	39.38;45.99;20.26
2015-TE-29068-1-1.fasta	Chicken food	Chicken	I	.	100	100	100	100	100	.
2015-TE-29222.fasta	Chicken food	Chicken	I	.	.	100	99.9	100	100	99.9
2015-TE-29232.fasta	Chicken food	Chicken	I	.	100	100	99.9	100	100	99.9
2015-TE-29243.fasta	Chicken food	Chicken	I	.	100	100	99.9	100	100	100
2015-TE-29919.fasta	Chicken animals	Chicken	I	.	100	100	100	100	100	.
2015-TE-29920.fasta	Chicken food	Chicken	I	.	100	100	100	100	100	99.9
2015-TE-29928.fasta	Chicken animals	Chicken	T	.	100	100	100	100	100	.
2015-TE-29930.fasta	Chicken food	Chicken	T	.	100	100	100	100	100	.

2015-TE-29940.fasta	Chicken animals	Chicken	T	.	100	100	100	100	100	.
2015-TE-29958.fasta	Chicken food	Chicken	I	100	.	100	99.9	100	100	99.9
2015-TE-29967-1-1.fasta	Crow	Urban_bird	T	86.88;10.17	.	100	100	100	100	100
2015-TE-29969-1-1.fasta	pigeons	Urban_bird	T	99.6	.	100	99.9	100	100	.
2015-TE-29984.fasta	Chicken food	Chicken	I	100	.	100	99.9	100	100	99.9
2015-TE-29992-1-1.fasta	Chicken food	Chicken	I	.	100	100	100	100	100	.
2015-TE-29993-1-1.fasta	Chicken food	Chicken	T	.	100	100	100	100	100	.
2015-TE-29994-1-1.fasta	Chicken food	Chicken	I	.	100	99.91	99.55	99.93	100	100
2015-TE-29995-1-1.fasta	Chicken food	Chicken	I	100	.	100	100	100	100	.
2015-TE-30001-1-1.fasta	pigeons	Urban_bird	T	97.19	.	100	100	100	100	.
2015-TE-30018.fasta	Chicken food	Chicken	I	100	.	99.91	99.55	100	100	100
2015-TE-30024.fasta	Chicken food	Chicken	I	.	100	100	100	100	100	99.9
2015-TE-30028.fasta	Chicken food	Chicken	I	.	.	100	99.9	100	100	99.9
2015-TE-30051.fasta	Chicken animals	Chicken	I	.	100	100	100	100	100	99.9
2015-TE-30052.fasta	Chicken animals	Chicken	I	100	.	100	99.9	100	100	99.9
2015-TE-30056.fasta	Chicken animals	Chicken	I	100	.	99.91	99.55	99.93	100	100
2015-TE-30060.fasta	Chicken animals	Chicken	I	.	100	100	100	100	100	99.9
2015-TE-30063.fasta	Chicken animals	Chicken	T	.	100	100	99.55	100	100	100
2015-TE-30072.fasta	Chicken animals	Chicken	I	.	100	100	100	100	100	99.9
2015-TE-30076.fasta	Chicken animals	Chicken	I	100	.	99.91	99.55	99.93	100	100
2015-TE-30081.fasta	Chicken animals	Chicken	I	100	.	99.91	99.55	99.93	100	100
2015-TE-30404.fasta	Chicken food	Chicken	I	.	100	100	99.9	100	100	99.9
2015-TE-30407.fasta	Chicken food	Chicken	I	.	100	100	100	100	100	.
2015-TE-30414.fasta	Chicken food	Chicken	I	.	100	100	99.9	100	100	99.9
2015-TE-30420.fasta	Chicken food	Chicken	I	.	100	100	100	100	100	99.9
2015-TE-30429.fasta	Chicken food	Chicken	T	.	100	100	99.9	100	100	100
2015-TE-30430.fasta	Chicken animals	Chicken	T	.	100	100	99.9	100	100	100
2015-TE-30432.fasta	Chicken animals	Chicken	I	100	.	99.91	99.55	99.93	100	100
2015-TE-30433.fasta	Chicken food	Chicken	I	100	.	99.91	99.55	99.93	100	100
2015-TE-30439.fasta	Chicken food	Chicken	T	.	100	100	100	100	100	.
2015-TE-30440.fasta	Chicken animals	Chicken	T	.	100	100	100	100	100	.
2015-TE-30441.fasta	Chicken food	Chicken	T	.	100	100	100	100	100	.

2015-TE-30445.fasta	Chicken animals	Chicken	T	.	100	100	100	100	100	.
2015-TE-30450.fasta	Chicken food	Chicken	I	.	100	100	100	100	100	99.9
2015-TE-30452.fasta	Chicken animals	Chicken	I	.	100	100	100	100	100	.
2015-TE-30496.fasta	Chicken animals	Chicken	I	.	100	100	100	100	100	99.9
2015-TE-30498.fasta	Chicken animals	Chicken	I	.	100	100	100	100	100	99.9
2015-TE-30499.fasta	Chicken animals	Chicken	I	.	100	99.91	99.55	99.93	100	100
2015-TE-30778.fasta	Chicken animals	Chicken	I	.	100	100	99.9	100	100	99.9
2015-TE-30779.fasta	Chicken food	Chicken	T	.	100	100	99.9	100	100	.
2015-TE-30783.fasta	Chicken food	Chicken	T	.	100	100	99.9	100	100	.
2015-TE-30794.fasta	Chicken food	Chicken	I	.	100	100	100	100	100	99.9
2015-TE-30796.fasta	Chicken animals	Chicken	I	.	100	100	100	100	100	99.9
2015-TE-30797.fasta	Chicken food	Chicken	I	.	100	100	100	100	100	99.9
2015-TE-30798.fasta	Chicken animals	Chicken	I	100	.	100	99.9	100	100	100
2015-TE-30809.fasta	Chicken food	Chicken	I	.	100	100	100	100	100	99.9
2015-TE-30810.fasta	Chicken animals	Chicken	I	100	.	100	99.9	100	100	100
2015-TE-30817.fasta	Chicken animals	Chicken	I	.	100	100	100	100	100	100
2015-TE-30821.fasta	Chicken food	Chicken	I	.	100	100	99.9	100	100	99.9
2015-TE-30891.fasta	Chicken animals	Chicken	I	100	.	100	99.9	100	100	100
2015-TE-30898.fasta	Chicken animals	Chicken	I	.	100	100	100	100	100	99.9
2015-TE-30901.fasta	Chicken animals	Chicken	I	.	100	100	100	100	100	99.9
2015-TE-30902-1-1.fasta	Chicken food	Chicken	I	.	100	100	100	100	100	99.9
2015-TE-31196-1-1.fasta	Chicken food	Chicken	I	100	.	100	99.9	100	100	99.9
2015-TE-31197-1-1.fasta	Chicken food	Chicken	I	100	.	99.91	99.55	100	100	100
2015-TE-31318.fasta	Chicken animals	Chicken	I	.	100	100	100	100	100	99.9
2015-TE-31321.fasta	Chicken animals	Chicken	I	100	.	99.91	99.55	100	100	100
2015-TE-31327.fasta	Chicken animals	Chicken	I	.	100	100	100	100	100	99.9
2015-TE-31328.fasta	Chicken animals	Chicken	I	.	100	100	100	100	100	99.9
2015-TE-31436.fasta	Chicken animals	Chicken	I	.	100	100	100	100	100	99.9
2015-TE-31437.fasta	Chicken food	Chicken	I	.	100	100	100	100	100	99.9
2015-TE-31438.fasta	Chicken food	Chicken	I	.	100	100	100	100	100	.
2015-TE-31508-1-1.fasta	Chicken food	Chicken	I	.	100	100	100	100	100	100
2015-TE-31510-1-1.fasta	Chicken food	Chicken	I	.	100	100	100	100	100	99.9

2015-TE-31511-1-1.fasta	Chicken food	Chicken	I	100	.	99.91	99.55	99.93	100	100
2015-TE-31514-1-1.fasta	Chicken food	Chicken	I	.	100	100	99.9	100	100	100
2015-TE-31718-1-1.fasta	pigeons	Urban_bird	T	88.62	.	100	100	100	100	100
2015-TE-31719-1-1.fasta	pigeons	Urban_bird	T	88.62	.	100	100	100	100	100
2015-TE-31824-1-1.fasta	Chicken food	Chicken	I	.	100	100	100	100	100	99.9
2015-TE-32242-1-1.fasta	Chicken food	Chicken	I	100	.	100	100	100	100	.
2015-TE-32243-1-1.fasta	Chicken food	Chicken	I	.	100	100	99.9	100	100	100
2015-TE-32254-1-1.fasta	Chicken food	Chicken	I	100	.	99.91	99.55	99.93	100	100
2015-TE-32262-1-1.fasta	Chicken food	Chicken	T	.	100	100	100	100	100	.
2015-TE-32276-1-1.fasta	Chicken food	Chicken	T	.	100	100	100	100	100	.
2015-TE-32615-1-1.fasta	Chicken food	Chicken	T	.	100	100	99.9	100	100	99.9
2015-TE-32617-1-1.fasta	Chicken food	Chicken	I	.	100	100	99.9	100	100	100
2015-TE-32620-1-1.fasta	Chicken food	Chicken	I	.	100	100	99.55	99.93	100	.
2015-TE-32621-1-1.fasta	Chicken food	Chicken	T	97.19	.	100	99,9	100	100	.
2015-TE-32622-1-1.fasta	Chicken food	Chicken	T	.	100	100	100	100	100	.
2015-TE-32719.fasta	Chicken animals	Chicken	I	100	.	100	99.9	100	100	100
2015-TE-32739.fasta	Chicken animals	Chicken	I	100	.	99.91	99.55	100	100	99.9
2015-TE-32740.fasta	Chicken animals	Chicken	I	100	.	99.91	99.55	100	100	99.9
2015-TE-32741.fasta	Chicken animals	Chicken	I	100	.	100	99.9	100	100	100
2015-TE-32742.fasta	Chicken animals	Chicken	I	100	.	100	99.9	100	100	100
2015-TE-32743.fasta	Chicken animals	Chicken	I	100	.	100	99.9	100	100	100
2015-TE-33101.fasta	Chicken animals	Chicken	I	100	.	99.91	99.55	100	100	99.9
2015-TE-33154-1-1.fasta	Chicken food	Chicken	I	.	100	100	100	100	100	.
2015-TE-33155-1-1.fasta	Chicken food	Chicken	I	.	100	100	99.9	100	100	99.9
2015-TE-33156-1-1.fasta	Chicken food	Chicken	I	.	100	100	99.9	100	100	99.9
2015-TE-33159-1-1.fasta	Chicken food	Chicken	I	.	100	100	100	100	100	99.9
2015-TE-33163.fasta	Chicken food	Chicken	I	100	.	99.91	99.55	99.93	100	100
2015-TE-33167.fasta	Chicken food	Chicken	I	100	.	99.91	99.55	99.93	100	100
2015-TE-33233-1-1.fasta	Chicken food	Chicken	I	.	100	100	100	100	100	.
2015-TE-33236.fasta	Chicken food	Chicken	I	.	100	100	100	100	100	99.9
2015-TE-33240.fasta	Chicken food	Chicken	I	.	100	100	100	100	100	.
2015-TE-33241.fasta	Chicken animals	Chicken	I	100	.	99.91	99.55	99.93	100	100

2015-TE-33243.fasta	Chicken food	Chicken	I	.	100	100	100	100	100	.
2015-TE-33589-1-1.fasta	Chicken food	Chicken	I	.	100	100	100	100	100	99.9
2015-TE-33718-1-1.fasta	Chicken food	Chicken	I	.	.	100	100	100	100	.
2015-TE-33720-1-1.fasta	Chicken food	Chicken	I	.	100	100	100	100	100	99.9
2015-TE-33722-1-1.fasta	Chicken food	Chicken	I	100	.	100	99.9	100	100	100
2015-TE-33723-1-1.fasta	Chicken food	Chicken	I	.	.	100	99.9	100	100	99.9
2015-TE-33724-1-1.fasta	Chicken food	Chicken	I	.	100	100	99.9	100	100	99.9
2015-TE-33727-1-1.fasta	Chicken food	Chicken	I	.	100	100	99.9	100	100	100
2015-TE-33728-1-1.fasta	Chicken food	Chicken	I	.	.	100	99.9	100	100	99.9
2015-TE-33729-1-1.fasta	Chicken food	Chicken	I	.	.	100	100	100	100	.
2015-TE-33733.fasta	Chicken animals	Chicken	I	100	.	100	99.9	100	100	100
2015-TE-33740-1-1.fasta	pigeons	Urban_bird	T	88.62	.	100	100	100	100	10.94
2015-TE-33856-1-1.fasta	Chicken food	Chicken	I	.	100	100	100	100	100	15.36;23.39;17.40
2015-TE-33858-1-1.fasta	Chicken food	Chicken	I	.	100	100	100	100	100	99.9
2015-TE-33859-1-1.fasta	Chicken food	Chicken	I	100	.	100	99.9	100	100	100
2015-TE-7900.fasta	human	Human	I	.	.	100	99.9	100	100	.
2015-TE-8887.fasta	Chicken food	Chicken	I	100	.	100	100	100	100	.
2015-TE-8888.fasta	Chicken animals	Chicken	I	.	.	100	99.9	100	100	99.9
2015-TE-9163.fasta	Chicken food	Chicken	T	.	100	100	99.9	100	100	.
2015-TE-9178.fasta	Chicken animals	Chicken	I	.	100	100	100	100	100	99.9
2015-TE-9523.fasta	Chicken food	Chicken	I	100	.	99.91	99.55	99.93	100	99.9
2015-TE-9530.fasta	Chicken animals	Chicken	I	.	100	100	100	100	100	99.9
2015-TE-9549.fasta	Chicken food	Chicken	I	.	100	100	100	100	100	99.9
2015-TE-9553.fasta	Chicken food	Chicken	I	.	100	100	100	100	100	99.9
2015-TE-9841.fasta	Chicken animals	Chicken	I	100	.	100	99.9	100	100	100
2015-TE-9842.fasta	Chicken animals	Chicken	I	100	.	100	99.9	100	100	100
2016-TE-10005-1-1.fasta	Chicken food	Chicken	I	.	100	100	100	100	100	99.9
2016-TE-1005.fasta	human	Human	T	.	100	100	99.9	100	100	.
2016-TE-1008.fasta	human	Human	I	.	100	99.91	99.55	99.93	100	.
2016-TE-1017.fasta	Chicken animals	Chicken	I	.	100	100	99.9	100	100	99.9
2016-TE-1021.fasta	human	Human	I	.	100	100	100	100	100	.
2016-TE-1022.fasta	human	Human	I	.	.	99.91	99.55	99.93	100	.

2016-TE-1025.fasta	human	Human	I	.	.	99.91	99.55	99.93	100	99.9
2016-TE-1027.fasta	Chicken animals	Chicken	I	.	100	100	100	100	100	99.9
2016-TE-1028-1-1.fasta	Chicken food	Chicken	I	.	100	100	100	100	100	.
2016-TE-10309-1-1.fasta	pigeons	Urban_bird	T	100	.	100	99.9	100	100	.
2016-TE-10310-1-1.fasta	pigeons	Urban_bird	T	99.6	.	100	99.9	100	100	.
2016-TE-10404.fasta	Chicken animals	Chicken	I	100	.	100	99.9	100	100	100
2016-TE-10405.fasta	Chicken animals	Chicken	I	100	.	100	99.9	100	100	100
2016-TE-10406.fasta	Chicken animals	Chicken	I	100	.	100	99.9	100	100	100
2016-TE-1043.fasta	human	Human	T	.	.	100	100	100	100	.
2016-TE-1045.fasta	Chicken animals	Chicken	I	.	100	100	100	100	100	99.9
2016-TE-1056.fasta	Chicken animals	Chicken	I	100	.	100	99.9	100	100	100
2016-TE-10662.fasta	Chicken animals	Chicken	T	97.19	.	100	99,9	100	100	.
2016-TE-10663.fasta	Chicken animals	Chicken	T	97.19	.	100	99.9	100	100	.
2016-TE-10664.fasta	Chicken animals	Chicken	T	97.19	.	100	99,9	100	100	.
2016-TE-1072-1-1.fasta	Chicken food	Chicken	I	100	.	100	99.9	100	100	100
2016-TE-1073-1-1.fasta	Chicken food	Chicken	I	.	100	100	100	100	100	99.9
2016-TE-1075-1-1.fasta	Chicken food	Chicken	I	.	100	100	100	100	100	.
2016-TE-1076-1-1.fasta	Chicken food	Chicken	I	.	.	100	100	100	100	.
2016-TE-10782-1-1.fasta	Chicken food	Chicken	I	.	100	100	100	100	100	.
2016-TE-10783-1-1.fasta	Chicken food	Chicken	I	.	100	100	100	100	100	99.9
2016-TE-10784-1-1.fasta	Chicken food	Chicken	I	.	100	100	100	100	100	99.9
2016-TE-10785-1-1.fasta	Chicken food	Chicken	I	.	100	100	100	100	100	.
2016-TE-10787-1-1.fasta	Chicken food	Chicken	I	100	.	100	99.9	100	100	100
2016-TE-10838.fasta	Chicken food	Chicken	T	97.19	.	100	99.9	100	100	.
2016-TE-10839.fasta	Chicken food	Chicken	T	97.19	.	100	99.9	100	100	.
2016-TE-10840.fasta	Chicken food	Chicken	T	97.19	.	100	99.9	100	100	.
2016-TE-10879-1-1.fasta	Chicken food	Chicken	T	.	100	100	100	100	100	.
2016-TE-1111.fasta	Chicken animals	Chicken	I	.	100	100	100	100	100	99.9
2016-TE-1117.fasta	Chicken animals	Chicken	I	.	100	100	100	100	100	99.9
2016-TE-1118.fasta	Chicken animals	Chicken	I	.	100	100	100	100	100	99.9
2016-TE-1124.fasta	Chicken animals	Chicken	I	100	.	99.91	99.55	99.93	100	100
2016-TE-1132.fasta	Chicken animals	Chicken	I	.	100	100	100	100	100	.

2016-TE-1133.fasta	Chicken animals	Chicken	T	.	.	100	99.9	100	100	99.9
2016-TE-12094-1-1.fasta	pigeons	Urban_bird	I	100	.	100	100	100	100	.
2016-TE-12096-1-1.fasta	pigeons	Urban_bird	T	99.6	.	100	99.9	100	100	.
2016-TE-12097-1-1.fasta	pigeons	Urban_bird	T	100	.	100	99.9	100	100	.
2016-TE-12389-1-1.fasta	Chicken food	Chicken	I	.	100	32.49;72.40	100	100	100	99.9
2016-TE-1281-1-1.fasta	Chicken food	Chicken	I	.	100	100	100	100	100	.
2016-TE-12816-1-1.fasta	pigeons	Urban_bird	T	99.6	.	100	99.9	100	100	.
2016-TE-1282-1-1.fasta	Chicken food	Chicken	T	.	100	99.91	99.55	100	100	100
2016-TE-13898-1-1.fasta	Chicken food	Chicken	I	100	.	100	99.9	100	100	100
2016-TE-13899-1-1.fasta	Chicken food	Chicken	I	100	.	99.91	99.55	99.93	100	100
2016-TE-1479-1-1.fasta	Chicken food	Chicken	I	.	100	100	100	100	100	.
2016-TE-1480-1-1.fasta	Chicken food	Chicken	I	.	100	100	100	100	100	99.9
2016-TE-1483-1-1.fasta	Chicken food	Chicken	I	100	.	100	99.9	100	100	100
2016-TE-1484-1-1.fasta	Chicken food	Chicken	I	.	100	100	100	100	100	99.9
2016-TE-1486-1-1.fasta	Chicken food	Chicken	I	.	100	100	100	100	100	99.9
2016-TE-15492.fasta	Chicken food	Chicken	I	100	.	99.91	99.55	99.93	100	100
2016-TE-15494-1-1.fasta	Chicken food	Chicken	T	.	100	100	100	100	100	.
2016-TE-15495-1-1.fasta	Chicken food	Chicken	I	.	100	100	100	100	100	100
2016-TE-15496-1-1.fasta	Chicken food	Chicken	I	.	100	100	99.9	100	100	.
2016-TE-15498-1-1.fasta	Chicken food	Chicken	I	.	100	100	100	100	100	99.9
2016-TE-15499-1-1.fasta	Chicken food	Chicken	I	.	100	100	100	100	100	99.9
2016-TE-15801-1-1.fasta	Chicken food	Chicken	I	.	.	100	100	100	100	.
2016-TE-16009-1-1.fasta	Chicken food	Chicken	I	.	100	100	100	100	100	100
2016-TE-16013-1-1.fasta	Chicken food	Chicken	I	100	.	100	99.9	100	100	100
2016-TE-16018-1-1.fasta	Chicken food	Chicken	I	.	100	100	100	100	100	99.9
2016-TE-16022-1-1.fasta	Chicken food	Chicken	I	.	100	100	100	100	100	99.9
2016-TE-16023-1-1.fasta	Chicken food	Chicken	I	.	100	100	100	100	100	99.9
2016-TE-1628-1-1.fasta	Chicken animals	Chicken	I	.	100	100	100	100	100	99.9
2016-TE-1630-1-1.fasta	Chicken animals	Chicken	I	.	100	100	100	100	100	.
2016-TE-1631-1-1.fasta	Chicken animals	Chicken	T	.	100	100	100	100	100	15.57;11.61;16.51
2016-TE-16559-1-1.fasta	Chicken food	Chicken	I	.	100	100	100	100	100	99.9
2016-TE-16560-1-1.fasta	Chicken food	Chicken	I	.	100	100	100	100	100	99.9

2016-TE-16561-1-1.fasta	Chicken food	Chicken	I	.	100	100	100	100	100	.
2016-TE-16562-1-1.fasta	Chicken food	Chicken	I	100	.	100	99.9	100	100	100
2016-TE-16564-1-1.fasta	Chicken food	Chicken	I	100	.	100	99.9	100	100	99.9
2016-TE-16565-1-1.fasta	Chicken food	Chicken	I	100	.	100	99.9	100	100	100
2016-TE-16566-1-1.fasta	Chicken food	Chicken	I	.	100	100	100	100	100	99.9
2016-TE-16567-1-1.fasta	Chicken food	Chicken	I	.	100	100	100	100	100	100
2016-TE-16568-1-1.fasta	Chicken food	Chicken	I	.	100	100	100	100	100	100
2016-TE-1682-1-1.fasta	Chicken animals	Chicken	I	.	100	100	100	100	100	99.9
2016-TE-1958-1-1.fasta	Chicken food	Chicken	I	.	100	99.91	99.55	100	100	100
2016-TE-1960-1-1.fasta	Chicken food	Chicken	I	.	100	100	100	100	100	99.9
2016-TE-1962-1-1.fasta	Chicken food	Chicken	I	.	100	100	100	100	100	.
2016-TE-20375-1-1.fasta	Crow	Urban_bird	T	97.19	.	100	100	100	100	.
2016-TE-20407-1-1.fasta	human	Human	I	100	.	100	99.9	100	100	100
2016-TE-22529-1-1.fasta	pigeons	Urban_bird	T	99.6	.	100	99.9	100	100	.
2016-TE-2288-1-1.fasta	Chicken food	Chicken	I	.	100	100	99.9	100	100	100
2016-TE-24037-1-1.fasta	pigeons	Urban_bird	T	99.6	.	100	99.9	100	100	.
2016-TE-24038-1-1.fasta	pigeons	Urban_bird	T	99.6	.	100	99.9	100	100	.
2016-TE-24471-1-1.fasta	Crow	Urban_bird	T	88.62	.	100	100	100	100	.
2016-TE-25584-1-1.fasta	Crow	Urban_bird	T	88.62	.	100	100	100	100	.
2016-TE-25585-1-1.fasta	pigeons	Urban_bird	T	99.6	.	100	99.9	100	100	.
2016-TE-25586-1-1.fasta	pigeons	Urban_bird	T	99.6	.	100	99.9	100	100	.
2016-TE-2659-1-1.fasta	Chicken animals	Chicken	I	100	.	100	99.9	100	100	100
2016-TE-2664-1-1.fasta	Chicken animals	Chicken	I	100	.	100	99.9	100	100	100
2016-TE-2665-1-1.fasta	Chicken animals	Chicken	I	100	.	100	99.9	100	100	100
2016-TE-27119-1-1.fasta	pigeons	Urban_bird	T	99.6	.	100	99.9	100	100	.
2016-TE-27121-1-1.fasta	pigeons	Urban_bird	T	99.6	.	100	99.9	100	100	.
2016-TE-27123-1-1.fasta	pigeons	Urban_bird	T	99.6	.	100	99.9	100	100	.
2016-TE-27124-1-1.fasta	pigeons	Urban_bird	T	99.6	.	100	99.9	100	100	.
2016-TE-27127-1-1.fasta	pigeons	Urban_bird	T	99.6	.	100	99.9	100	100	.
2016-TE-2793-1-1.fasta	Chicken food	Chicken	I	.	100	100	100	100	100	99.9
2016-TE-2986-1-1.fasta	Chicken animals	Chicken	V	.	100	100	100	100	100	100
2016-TE-2989-1-1.fasta	Chicken animals	Chicken	I	.	100	100	100	100	100	.

2016-TE-2994-1-1.fasta	Chicken animals	Chicken	I	.	100	100	100	100	100	99.9
2016-TE-3000-1-1.fasta	Chicken food	Chicken	I	.	100	100	100	100	100	99.9
2016-TE-30153-1-1.fasta	Chicken food	Chicken	I	.	100	100	100	100	100	99.9
2016-TE-30158-1-1.fasta	human	Human	T	.	100	100	99.9	100	100	.
2016-TE-30159-1-1.fasta	human	Human	T	.	100	100	100	100	99.84	99.9
2016-TE-30160-1-1.fasta	human	Human	I	.	100	100	99.9	100	100	100
2016-TE-30161-1-1.fasta	human	Human	I	.	.	100	99.9	100	100	100
2016-TE-30162-1-1.fasta	human	Human	I	.	100	100	100	100	100	.
2016-TE-30164-1-1.fasta	human	Human	I	.	100	99.91	99.55	99.93	100	.
2016-TE-30165-1-1.fasta	human	Human	I	.	100	100	100	100	100	99.9
2016-TE-3239-1-1.fasta	Chicken animals	Chicken	I	.	100	100	100	100	100	99.9
2016-TE-3240-1-1.fasta	Chicken animals	Chicken	I	.	100	100	100	100	100	.
2016-TE-3259-1-1.fasta	Chicken animals	Chicken	I	.	100	100	100	100	100	.
2016-TE-3269-1-1.fasta	Chicken animals	Chicken	I	.	100	100	100	100	100	.
2016-TE-3270-1-1.fasta	Chicken animals	Chicken	I	100	.	100	99.9	99.93	100	100
2016-TE-3567-1-1.fasta	Chicken food	Chicken	T	.	100	100	99.9	100	100	.
2016-TE-3568-1-1.fasta	Chicken food	Chicken	I	.	100	100	100	100	100	99.9
2016-TE-3570-1-1.fasta	Chicken food	Chicken	I	.	100	100	100	100	100	.
2016-TE-3576-1-1.fasta	Chicken food	Chicken	I	.	100	100	100	100	100	.
2016-TE-3577-1-1.fasta	Chicken food	Chicken	I	.	100	100	100	100	100	.
2016-TE-3578-1-1.fasta	Chicken food	Chicken	I	100	.	100	99.9	100	100	100
2016-TE-4129-1-1.fasta	Chicken animals	Chicken	I	.	100	100	100	100	100	99.9
2016-TE-4135-1-1.fasta	Chicken animals	Chicken	I	.	100	100	100	100	100	99.9
2016-TE-4140-1-1.fasta	Chicken food	Chicken	I	.	100	100	100	100	100	.
2016-TE-4143-1-1.fasta	Chicken animals	Chicken	I	.	100	100	100	100	100	.
2016-TE-4144-1-1.fasta	Chicken animals	Chicken	I	.	100	99.91	99.55	99.93	100	100
2016-TE-4613-1-1.fasta	Chicken food	Chicken	I	.	100	100	100	100	100	99.9
2016-TE-4698.fasta	Chicken animals	Chicken	I	100	.	100	100	100	100	.
2016-TE-4700.fasta	Chicken animals	Chicken	I	100	.	100	100	100	100	.
2016-TE-4702.fasta	Chicken animals	Chicken	I	100	.	100	100	100	100	.
2016-TE-4703.fasta	Chicken animals	Chicken	I	100	.	100	100	100	100	.
2016-TE-4704.fasta	Chicken animals	Chicken	I	100	.	100	100	100	100	.

2016-TE-4706.fasta	Chicken animals	Chicken	I	100	.	100	100	100	100	.
2016-TE-4754-1-1.fasta	Chicken animals	Chicken	I	.	100	100	99.9	100	100	100
2016-TE-4756-1-1.fasta	Chicken animals	Chicken	I	100	.	100	99.9	99.93	100	100
2016-TE-4761-1-1.fasta	Chicken animals	Chicken	I	100	.	100	99.9	99.93	100	100
2016-TE-4764-1-1.fasta	Chicken animals	Chicken	I	.	100	100	99.9	100	100	100
2016-TE-4765-1-1.fasta	Chicken animals	Chicken	I	100	.	99.91	99.55	99.93	100	39.38;45.99;20.26
2016-TE-4766-1-1.fasta	Chicken animals	Chicken	I	100	.	100	99.9	100	100	100
2016-TE-4771-1-1.fasta	Chicken animals	Chicken	T	.	100	99.91	99.55	100	100	100
2016-TE-4777-1-1.fasta	Chicken animals	Chicken	I	.	100	100	100	100	100	99.9
2016-TE-4797-1-1.fasta	Chicken animals	Chicken	I	.	100	100	99.9	100	100	100
2016-TE-4804-1-1.fasta	Chicken animals	Chicken	T	.	100	100	100	100	100	99.9
2016-TE-4806-1-1.fasta	Chicken animals	Chicken	I	.	100	100	100	100	100	99.9
2016-TE-4808-1-1.fasta	Chicken animals	Chicken	I	.	100	99.91	99.55	100	100	100
2016-TE-4810-1-1.fasta	Chicken animals	Chicken	T	100	.	100	99.9	100	100	99.9
2016-TE-4811-1-1.fasta	Chicken animals	Chicken	T	.	100	100	99.9	100	100	.
2016-TE-4819-1-1.fasta	Chicken animals	Chicken	I	100	.	100	99.9	100	100	100
2016-TE-6009-1-1.fasta	pigeons	Urban_bird	T	97.19	.	100	100	100	100	23.75
2016-TE-6010-1-1.fasta	pigeons	Urban_bird	T	97.19	.	100	100	100	100	.
2016-TE-6262.fasta	Chicken animals	Chicken	I	.	7.11;56.33;50.78	100	100	100	100	.
2016-TE-6264.fasta	Chicken animals	Chicken	T	100	100	100	100	100	100	100
2016-TE-6265.fasta	Chicken animals	Chicken	I	16.73;69.21	100	100	100	100	100	15.78;27.92
2016-TE-6448-1-1.fasta	Chicken animals	Chicken	T	.	100	100	100	100	100	99.9
2016-TE-6463-1-1.fasta	Chicken animals	Chicken	I	.	100	100	100	100	100	99.9
2016-TE-6832-1-1.fasta	pigeons	Urban_bird	T	99.6	.	100	99.9	100	100	.
2016-TE-6834-1-1.fasta	pigeons	Urban_bird	T	99.6	.	100	99.9	100	100	.
2016-TE-7428-1-1.fasta	Chicken food	Chicken	T	.	100	100	99.9	100	100	100
2016-TE-7430-1-1.fasta	Chicken food	Chicken	I	100	.	100	99.9	100	100	100
2016-TE-7431-1-1.fasta	Chicken food	Chicken	I	100	.	100	99.9	100	100	100
2016-TE-7432-1-1.fasta	Chicken food	Chicken	I	.	100	100	100	100	100	.
2016-TE-7433-1-1.fasta	Chicken food	Chicken	T	.	100	100	100	100	100	.
2016-TE-7435-1-1.fasta	Chicken food	Chicken	I	.	100	100	100	100	100	99.9
2016-TE-7439-1-1.fasta	Chicken food	Chicken	I	.	100	100	99.9	100	100	100

2016-TE-7440-1-1.fasta	Chicken food	Chicken	I	100	.	100	99.9	100	100	100
2016-TE-7816-1-1.fasta	Chicken food	Chicken	I	.	100	100	100	100	100	.
2016-TE-7817-1-1.fasta	Chicken food	Chicken	I	100	.	100	99.9	100	100	100
2016-TE-7822-1-1.fasta	Chicken food	Chicken	V	.	100	100	100	100	100	100
2016-TE-7827-1-1.fasta	Chicken food	Chicken	I	97.19	.	100	99.9	100	100	99.9
2016-TE-8161-1-1.fasta	Chicken food	Chicken	I	.	100	100	100	100	100	99.9
2016-TE-8199-1-1.fasta	pigeons	Urban_bird	T	97.19	.	100	100	100	100	.
2016-TE-8476-1-1.fasta	Chicken food	Chicken	I	100	.	100	99.9	100	100	100
2016-TE-8482-1-1.fasta	Chicken food	Chicken	T	.	100	100	99.9	100	100	.
2016-TE-8485-1-1.fasta	Chicken food	Chicken	V	.	100	100	100	100	100	100
2016-TE-8487-1-1.fasta	Chicken food	Chicken	T	.	100	100	100	100	100	.
2016-TE-8488-1-1.fasta	Chicken food	Chicken	T	.	100	100	100	100	100	99.9
2016-TE-8489-1-1.fasta	Chicken food	Chicken	I	100	.	99.91	99.55	99.93	100	100
2016-TE-8490-1-1.fasta	Chicken food	Chicken	V	.	100	100	100	100	100	100
2016-TE-8491-1-1.fasta	Chicken food	Chicken	I	100	.	100	99.9	100	100	99.9
2016-TE-8593-1-1.fasta	Chicken food	Chicken	I	.	100	100	100	100	100	99,9
2016-TE-8597-1-1.fasta	pigeons	Urban_bird	T	99.6	.	100	99.9	100	100	.
2016-TE-895.fasta	human	Human	I	.	.	100	100	100	100	100
2016-TE-8991-1-1.fasta	Chicken food	Chicken	I	.	100	100	100	100	100	99.9
2016-TE-8993-1-1.fasta	Chicken food	Chicken	I	.	100	100	100	100	100	99.9
2017.TE.27617.1.1.fasta	pigeons	Urban_bird	I	.	100	100	100	100	100	99.9
2017.TE.27619.1.1.fasta	pigeons	Urban_bird	I	98.13	.	100	99.9	100	100	.
2017.TE-27620.1.1.fasta	pigeons	Urban_bird	T	99.6	.	100	99.9	24.48;100.00	100	.
2017-TE-12276-1-1.fasta	pigeons	Urban_bird	T	88.62	.	100	100	100	100	100
2017-TE-12278-1-1.fasta	magpie	Urban_bird	T	.	100	100	99.9	100	99.84	.
2017-TE-12279-1-1.fasta	pigeons	Urban_bird	T	99.6	.	100	99.9	100	100	.
2017-TE-13762.fasta	human	Human	T	.	.	100	100	100	100	.
2017-TE-13763.fasta	human	Human	I	.	100	100	100	100	100	100
2017-TE-13764.fasta	human	Human	I	97.32	.	100	99,9	100	100	.
2017-TE-13765.fasta	human	Human	I	.	100	100	100	100	100	.
2017-TE-13766.fasta	human	Human	I	.	100	100	100	100	100	99.9
2017-TE-13767.fasta	human	Human	I	.	100	100	100	100	100	.

2017-TE-13768.fasta	human	Human	I	100	.	100	99.9	100	100	100
2017-TE-13769.fasta	human	Human	I	100	.	100	99.9	100	100	100
2017-TE-13771.fasta	human	Human	I	.	100	100	100	100	100	45.99;39.38;22.66
2017-TE-13772.fasta	human	Human	I	.	100	100	100	100	100	45.99;39.38;22.66
2017-TE-13775.fasta	human	Human	I	100	.	100	99.9	100	100	100
2017-TE-13781.fasta	human	Human	T	.	100	100	100	100	100	.
2017-TE-13782.fasta	human	Human	T	.	100	100	100	100	98.42	.
2017-TE-13783.fasta	human	Human	I	.	100	100	99.9	100	100	99.9
2017-TE-13784.fasta	human	Human	T	.	100	100	100	100	100	.
2017-TE-13785.fasta	human	Human	I	.	100	100	100	100	100	100
2017-TE-13787.fasta	human	Human	T	.	100	100	100	100	99.84	99.9
2017-TE-13788.fasta	human	Human	I	.	100	100	100	100	100	.
2017-TE-13789.fasta	human	Human	I	.	100	100	99.9	100	100	100
2017-TE-13790.fasta	human	Human	I	.	100	100	100	100	100	9.06;45.99;39.38;22.55
2017-TE-13791.fasta	human	Human	I	.	100	100	99.9	100	100	99.9
2017-TE-13793.fasta	human	Human	T	.	100	99.91	99.55	100	100	100
2017-TE-13795.fasta	human	Human	I	.	100	100	99.9	100	100	99.9
2017-TE-13796.fasta	human	Human	I	.	100	100	99.9	100	100	99.9
2017-TE-13797.fasta	human	Human	I	.	100	100	100	100	100	99.9
2017-TE-13798.fasta	human	Human	I	.	100	100	100	100	100	.
2017-TE-16477-1-1.fasta	pheasant	Urban_bird	T	.	100	100	100	99.93	97	99.9
2017-TE-16478-1-1.fasta	pigeons	Urban_bird	T	99.6	.	100	99.9	100	100	.
2017-TE-16479-1-1.fasta	pigeons	Urban_bird	T	99.6	.	100	99.9	100	100	.
2017-TE-16480-1-1.fasta	pigeons	Urban_bird	T	99.6	.	100	99.9	100	100	.
2017-TE-16481-1-1.fasta	pigeons	Urban_bird	T	99.6	.	100	99.9	100	100	.
2017-TE-17866-1-1.fasta	mallard	Wild_bird	T	100	.	100	100	100	100	.
2017-TE-17868-1-1.fasta	mallard	Wild_bird	T	100	.	100	99.9	100	100	.
2017-TE-19477-1-1.fasta	pigeons	Urban_bird	T	99.6	.	100	99.9	100	100	.
2017-TE-19479-1-1.fasta	pigeons	Urban_bird	T	99.6	.	100	99.9	100	100	.
2017-TE-19481-1-1.fasta	pigeons	Urban_bird	T	99.6	.	100	99.9	100	100	.
2017-TE-19486-1-1.fasta	pigeons	Urban_bird	T	99.6	.	100	99.9	100	100	.
2017-TE-19487-1-1.fasta	pigeons	Urban_bird	T	99.6	.	100	99.9	100	100	.

2017-TE-3069-1-1.fasta	pigeons	Urban_bird	T	99.6	.	100	99.9	100	100	.
2017-TE-4824-1-1.fasta	pigeons	Urban_bird	T	99.6	.	100	100	100	100	.
2017-TE-6838-1-1.fasta	pigeons	Urban_bird	T	.	.	100	99.9	100	100	99.9
2017-TE-6840-1-1.fasta	pigeons	Urban_bird	T	99.6	.	100	99.9	100	100	.
2017-TE-6841-1-1.fasta	pigeons	Urban_bird	T	99.6	.	100	99.9	100	100	.
